# Supplementary material for: A phase 1 study of nivolumab in combination with interferon-gamma for patients with advanced solid tumors
Source: Nat Commun. 2023 Jul 27;14:4513. doi: 10.1038/s41467-023-40028-z (PMC10374608; doi:10.1038/s41467-023-40028-z)
Supplement: Supplementary file 1 — Supplementary Information [file 41467_2023_40028_MOESM1_ESM.pdf]

Supplementary Information

A Phase 1 Study of Nivolumab in Combination with Interferon-Gamma for Patients with Advanced Solid Tumors

Supplementary Figure 1: Progression Free Survival Curve for all Evaluable Patients

Kaplan-Meier curve of progression free survival (PFS) of all patients evaluable for efficacy on study. Median PFS by this method is shown. Source data are provided as a Source Data file.

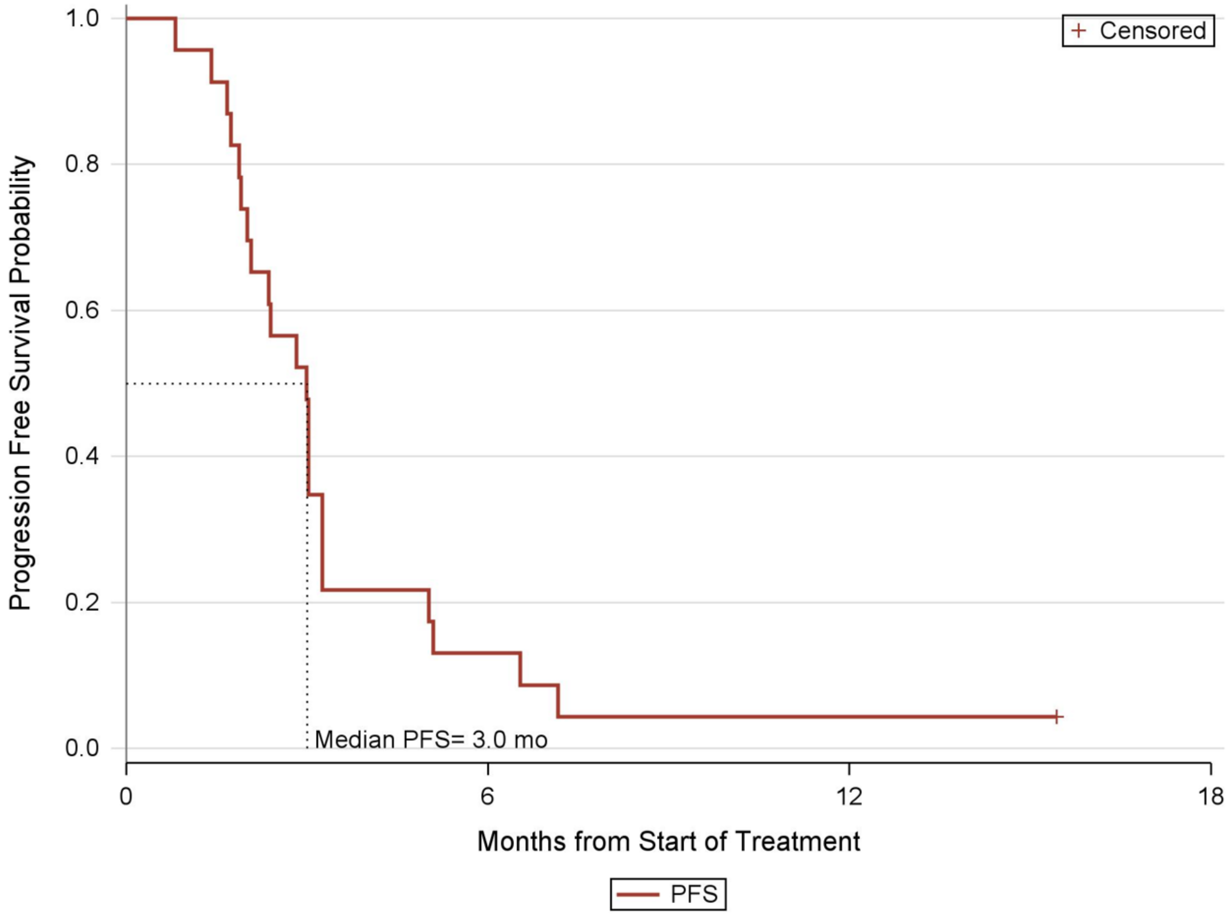

Supplementary Figure 2: Overall Survival Curve for all Evaluable Patients

Kaplan-Meier curve of overall survival (OS) of all patients evaluable for efficacy on study. Median OS by this method is shown. Source data are provided as a Source Data file.

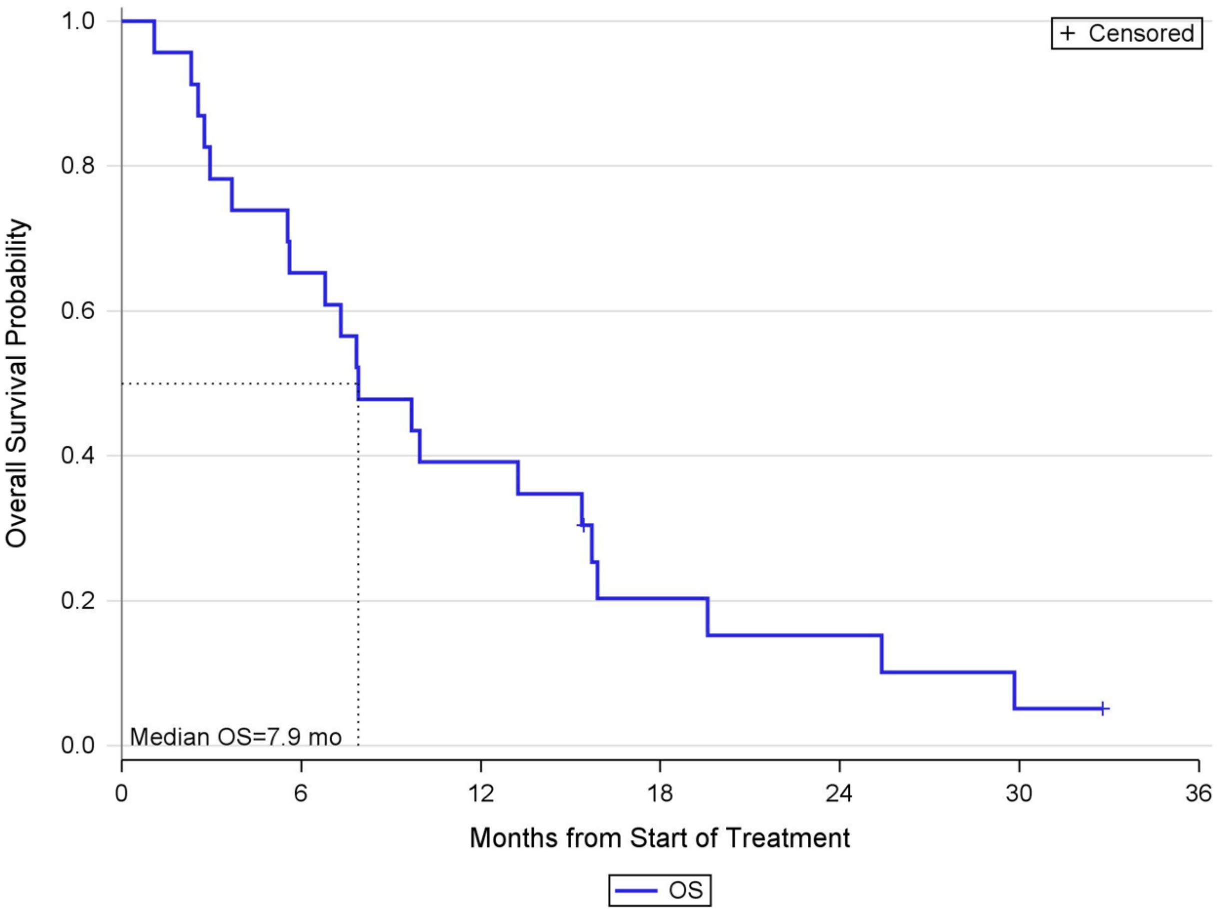

Supplementary Figure 3: Changes in additional chemokines/cytokines after induction and addition of nivolumab

Data from n=25 patients were used for the baseline vs. C1D1 comparison and n=20 patients were used for the baseline vs. C2D15 comparison for the following markers: CCL2, CCL23, CCL24, CCL26, CXCL13, CX3CL1 and IFNgamma. Data from n=23 patients were used for the baseline vs. C1D1 comparison and n=18 patients were used for the baseline vs. C2D15 comparison for GMCSF. Data from n=24 patients were used for the baseline vs. C1D1 comparison and n=20 patients were used for the baseline vs. C2D15 comparison for IL4.

The complete data set was assessed for two statistical comparisons (baseline vs. C1D1 and baseline vs C2D15). The two-sided Wilcoxon test was used to compare chemokine/cytokine levels in each case. In order to account for multiple hypotheses, the Benjamini-Hochberg false discovery rate (FDR) was computed, separately, for each of the different slices per comparison (pair of time-points) listed above. In addition to the data shown in Figure 4, any other changes in concentrations of individual chemokines and cytokines from baseline levels that were statistically significant at FDR$\leq$ 0.05 are shown and marked with an asterisk. Box-plots were plotted using default settings in the R language [1]. In each boxplot, the bold line in the center of the box indicates the median; and the lower and upper hinges represent, respectively, the first and third quartiles. The whiskers are computed based on 1.5 times the inter-quartile range (IQR). They extend to the most extreme data point which is no more than 1.5 times the IQR away from the box. If no points exceed this distance, then the whiskers are the minimum and maximum values. If there are points beyond that distance, then the most extreme point that does not exceed this distance is the whisker. Any data points shown beyond the whiskers are considered outliers. Source data are provided as a Source Data file.

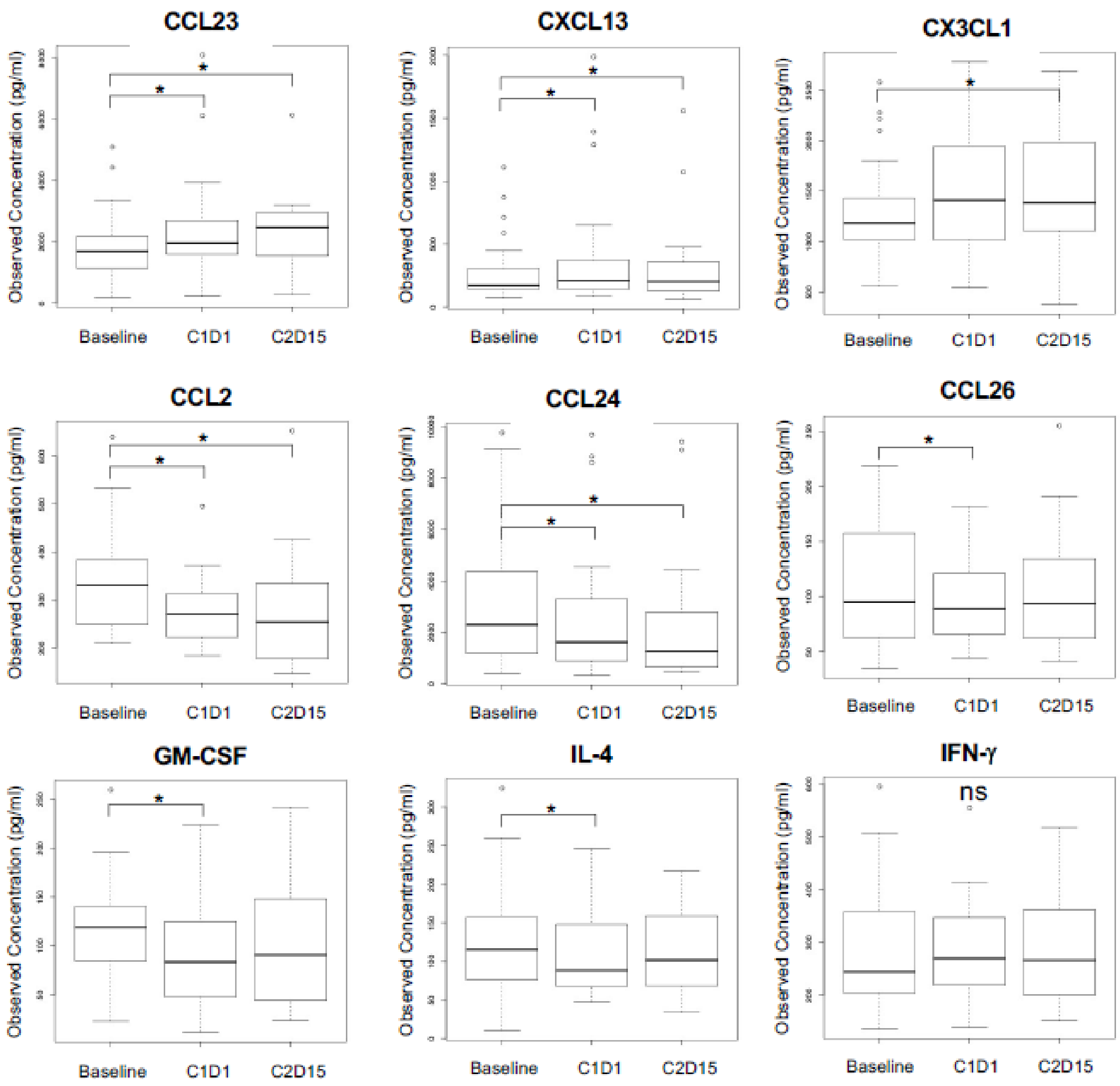

CCL23
CXCL13
CX3CL1
CCL2
CCL24
CCL26
GM-CSF
IL-4
IFN-γ
ns
Observed Concentration (pg/ml)
Baseline
C1D1
C2D15
*

Supplementary Figure 4. Monocyte Gating Strategy for Figure 2

A) Sequential gating of single cells, viable cells, leukocytes, and myeloid cells. The myeloid gate is pushed out slightly at high side scatter to capture CD56^{dim} monocytes. B) Monocytes are further defined as CD66b^{-} and CD11c^{+}. C) Developmental subsets are designated as Classical Monocytes (CM; CD14^{+} CD16^{-}), Intermediate Monocytes (IM; CD14^{+} CD16^{+}), and Non-classical Monocytes (NCM; CD14^{-} CD16^{+}). The Double Negative population (DN; CD14^{-} CD16^{-}) consists mostly of dendritic cells and is not described in this publication. Total monocytes are the combined Classical, Intermediate, and Non-classical subsets.

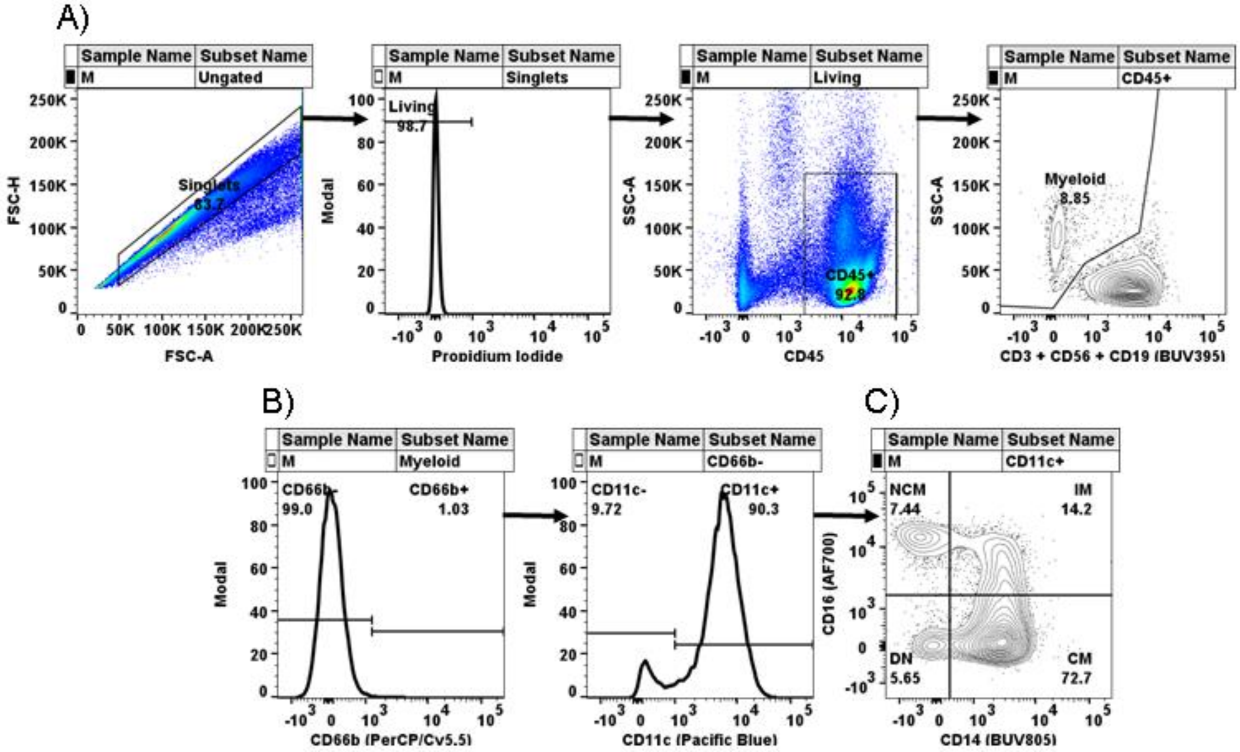

Supplementary Figure 5. T Cell Gating Strategy for Figure 3

A) Sequential gating of single cells, viable cells, lymphocytes, and T cells. B) Subsets of T cells based on CD4 and CD8 staining. C) Further subsetting of effector and memory cells designated as Naïve (N; CD45RA$^+$ CD62L$^+$), Central Memory (CM; CD45RA$^-$ CD62L$^+$), Effector (E; CD45RA$^+$ CD62L$^-$), and Effector Memory (EM; CD45RA$^-$ CD62L$^-$).

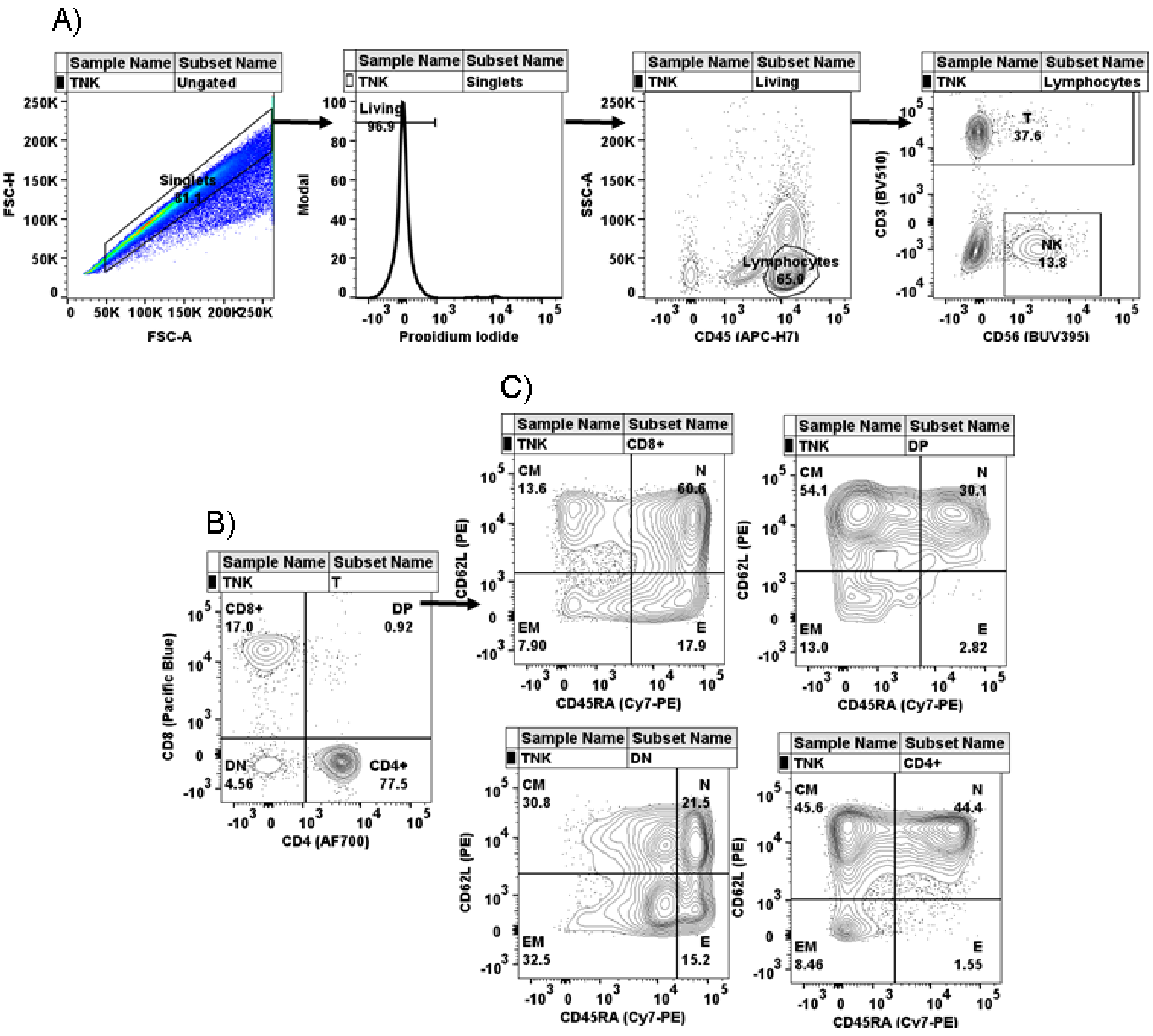

Supplementary Table 1: All-grade adverse events attributed as at least possibly related to either drug and stratified by dose level and grade (* denotes dose limiting toxicity)

	Any Grade 1 to 4	All, n=26		IFN 25 mcg/m^2, n=5		IFN 50 mcg/m^2, n=8		IFN 75 mcg/m^2, n=7		IFN 100 mcg/m^2, n=6	
		Grade		Grade		Grade		Grade		Grade	
**Adverse Event Term**	**n (%)**	**1-2**	**3+**	**1-2**	**3+**	**1-2**	**3+**	**1-2**	**3+**	**1-2**	**3+**
Fatigue	20 (76.9)	16	4	5		3	2	4	1*	4	1*
Fever	15 (57.7)	15		3		3		5		4	
Aspartate aminotransferase increased	14 (53.8)	13	1	2		4		6		1	1*
Myalgia	14 (53.8)	14		5		2		5		2	
Anorexia	13 (50.0)	13		2		3		3		5	
Chills	13 (50.0)	13		2		3		5		3	
Headache	13 (50.0)	13		2		4		5		2	
Alanine aminotransferase increased	11 (42.3)	11		1		3		4		3	
White blood cell decreased	9 (34.6)	8	1	1		1		5	1	1	
Anemia	8 (30.8)	6	2	2		1	1	2		1	1
Lymphocyte count decreased	8 (30.8)	7	1	1		3		2	1	1	
Arthralgia	7 (26.9)	7		2		1		2		2	
Weight loss	7 (26.9)	7				3		1		3	
Intermittent sweats	6 (23.1)	6		1		2		3			
Alkaline phosphatase increased	5 (19.2)	5		1		2				2	
Nausea	5 (19.2)	5				2		2		1	
Diarrhea	4 (15.4)	4				2		1		1	
Dyspnea	4 (15.4)	4		2				1		1	
Hot flashes	4 (15.4)	4		2				1		1	
Hyponatremia	4 (15.4)	2	2				1	1		1	1
Neutrophil count decreased	4 (15.4)	4						4			

	Any Grade 1 to 4	All, n=26		IFN 25 mcg/m^2, n=5		IFN 50 mcg/m^2, n=8		IFN 75 mcg/m^2, n=7		IFN 100 mcg/m^2, n=6	
		Grade		Grade		Grade		Grade		Grade	
**Adverse Event Term**	**n (%)**	**1-2**	**3+**	**1-2**	**3+**	**1-2**	**3+**	**1-2**	**3+**	**1-2**	**3+**
**Dizziness**	**3 (11.5)**	**3**				**1**		**2**			
**Pain in extremity**	**3 (11.5)**	**3**		**1**		**1**		**1**			
**Pleural effusion**	**3 (11.5)**	**1**	**2**		**1**					**1**	**1***
**Back pain**	**2 (7.7)**	**2**						**1**		**1**	
**Blood bilirubin increased**	**2 (7.7)**	**2**						**1**		**1**	
**Constipation**	**2 (7.7)**	**2**						**1**		**1**	
**Creatinine increased**	**2 (7.7)**	**2**				**1**				**1**	
**Edema limbs**	**2 (7.7)**	**2**		**1**						**1**	
**Hyperthyroidism**	**2 (7.7)**	**2**		**1**		**1**					
**Hypokalemia**	**2 (7.7)**	**2**				**1**				**1**	
**Hypothyroidism**	**2 (7.7)**	**2**				**1**				**1**	
**Insomnia**	**2 (7.7)**	**2**				**1**		**1**			
**Nail bed changes**	**2 (7.7)**	**2**				**1**		**1**			
**Blurred vision**	**1 (3.8)**	**1**						**1**			
**Cognitive disturbance**	**1 (3.8)**	**1**								**1**	
**Confusion**	**1 (3.8)**	**1**				**1**					
**Depression**	**1 (3.8)**	**1**						**1**			
**Dry eye**	**1 (3.8)**	**1**				**1**					
**Dry mouth**	**1 (3.8)**	**1**						**1**			
**Dysesthesia**	**1 (3.8)**	**1**						**1**			
**Dysphagia**	**1 (3.8)**	**1**						**1**			
**Flushing**	**1 (3.8)**	**1**				**1**					
**Generalized muscle weakness**	**1 (3.8)**	**1**				**1**					

	Any Grade 1 to 4	All, n=26		IFN 25 mcg/m^2, n=5		IFN 50 mcg/m^2, n=8		IFN 75 mcg/m^2, n=7		IFN 100 mcg/m^2, n=6	
		Grade		Grade		Grade		Grade		Grade	
**Adverse Event Term**	**n (%)**	**1-2**	**3+**	**1-2**	**3+**	**1-2**	**3+**	**1-2**	**3+**	**1-2**	**3+**
**Hallucinations**	**1 (3.8)**	**1**								**1**	
**Hypoalbuminemia**	**1 (3.8)**	**1**		**1**							
**Hypocalcemia**	**1 (3.8)**	**1**				**1**					
**Hypomagnesemia**	**1 (3.8)**	**1**				**1**					
**Hypophosphatemia**	**1 (3.8)**	**1**								**1**	
**Thrush**	**1 (3.8)**	**1**						**1**			
**Injection site reaction**	**1 (3.8)**	**1**						**1**			
**Irritability**	**1 (3.8)**	**1**						**1**			
**Hyperphosphatemia**	**1 (3.8)**	**1**						**1**			
**Neck edema**	**1 (3.8)**	**1**				**1**					
**Pain**	**1 (3.8)**	**1**						**1**			
**Palpitations**	**1 (3.8)**	**1**						**1**			
**Peripheral sensory neuropathy**	**1 (3.8)**	**1**				**1**					
**Platelet count decreased**	**1 (3.8)**	**1**								**1**	
**Pruritus**	**1 (3.8)**	**1**		**1**							
**Sinus tachycardia**	**1 (3.8)**	**1**		**1**							
**Skin infection**	**1 (3.8)**	**1**				**1**					
**Urinary tract infection**	**1 (3.8)**	**1**				**1**					
**Ventricular arrhythmia**	**1 (3.8)**	**1**								**1**	
**Ventricular tachycardia**	**1 (3.8)**	**1**		**1**							
**Vomiting**	**1 (3.8)**	**1**								**1**	

Supplementary Table 2: Adverse Events Attributable to IFN-gamma during Induction Phase

Adverse events (AEs) during one week single-agent induction phase of trial during which time patients received IFN-gamma alone. AEs are all grade 1-2 and are listed across the entire cohort and separated by dose level.

		IFN 25 mcg/m^2, n=5	IFN 50 mcg/m^2, n=8	IFN 75 mcg/m^2, n=7	IFN 100 mcg/m^2, n=6
**Adverse Event Term**	**n (%)**				
Chills	9 (34.6)	2	4		3
Myalgia	6 (23.1)	1	4		1
Fatigue	4 (15.4)	2	1	1	
Fever	4 (15.4)		3		1
Headache	4 (15.4)	2		1	1
Lymphocyte count decreased	2 (7.7)		2		
Flushing	2 (7.7)	1			1
Abdominal pain	1 (3.8)				1
Nausea	1 (3.8)	1			
General disorders and administration site conditions - Other	1 (3.8)	1			
Aspartate aminotransferase increased	1 (3.8)		1		
White blood cell decreased	1 (3.8)		1		
Anorexia	1 (3.8)		1		
Back pain	1 (3.8)				1
Cough	1 (3.8)				1
Hot flashes	1 (3.8)		1		

Supplementary Table 3: Individual Patient Accounting of Development of Ascites and Pleural Effusions on Treatment

In depth summary of each recorded episode of new pleural effusion or ascites while on treatment with IFN-gamma and/or nivolumab. The smaller table is separated into patients with new pleural effusions and new ascites, and presents the total number affected by dose level. The larger table summarizes these occurrences in each patient along with primary disease type, site of known metastases at baseline, grade of occurrence, drug attribution, whether it was counted as a DLT, and individual details if pertinent.

Pleural Effusion		Ascites	
Cohort #	# of Pts	Cohort #	# of Pts
1 (50 mcg/m^2)	1	1 (50 mcg/m^2)	1
2 (75 mcg/m^2)	0	2 (75 mcg/m^2)	2
3 (100 mcg/m^2)	3	3 (100 mcg/m^2)	1
4 (25 mcg/m^2)	1	4 (25 mcg/m^2)	1
**Total**	**5**	**Total**	**5**

Cohort	Onset Cycle	Onset Day	Cancer	Sites of Mets	AE Term	Grade	Attribution	Drug	DLT	Serious	Action
1	2	1	Ovarian	Lymph node, liver, and lung	Ascites	2	Unrelated	N/A	No	No	None
1	1	1	Breast	Lungs, pleura, pericardium, peritoneum, and bone (T7-T8)	Pleural effusion	2	Unrelated	N/A	No	No	None
2	1	1	Ovarian	Peritoneal lymph nodes, retroperitoneal lymph nodes, right internal mammary	Ascites	2	Unrelated	N/A	No	No	None
3	2	1	Endometrial	B/L lung, B/L pleura, lymph nodes	Pleural effusion	3	Possible	Interferon Gamma 1 b; Nivolumab	Yes	No	Interrupted and not reduced. Off trial for worsening effusion possibly attributed to IFN however ultimately due to disease progression
3	2	1	Gastric	Ovary and abdomen	Pleural effusion	1	Possible	Interferon Gamma 1 b; Nivolumab	No	No	None
4	1	1	Kidney	Lung	Pleural effusion	2	Probable	Interferon Gamma 1 b	No	No	Off drug and trial due to effusion
4	1	1	Ovarian	Liver, lymph nodes, Lung, Peritoneum	Ascites	2	Possible	Interferon Gamma 1 b	No	No	Therapy discontinued

Supplementary Table 4. Flow Cytometry Antibody Staining Panel and Validation Links
Each column designates a staining tube with antibody target antigens, commercial sources with catalog numbers, hybridoma clones, and µL added to 100 µL of PBMC suspension listed. Conjugated fluorophore is listed in the far left column. BD = Becton Dickinson, R&D = R&D Systems, Southern Bio = Southern Biotechnology, Nivo = nivolumab, Ms = mouse.

	T/NK	Treg/NK	PD-1/NK	Myeloid	Cytoplasmic (Fix/Perm)	Isotype Control/NK	Isotype Control (Fix/Perm)
FITC	**DNAM-1** Biolegend #337104 Clone TX25 5 ul	**CD25** BD #555431 Clone M-A251 20 ul	**NKp80** Biolegend #346708 Clone 5D12 5 ul	**IL-15RA** eBioscience #11-7159-42 CloneeBioJM7 A4 5 ul	**Granzyme K** Santa Cruz #sc-56125 Clone GM6C3 5 ul	**Rat IgG2b** Biolegend #400634 Clone RTK 4530 5 ul	**Ms IgG2b** Santa Cruz #sc-2856 Clone 185-1G2 5 ul
PE	**CD62L** Biolegend #304806 Clone DREG-56 5 ul	**NKp44** Biolegend #325108 Clone P44-8 5 ul	**Nivo (1 ug) + hIgG4** Southern Bio #9200-09 Clone HP6025 10 ul	**Nivo (1 ug) + hIgG4** Southern Bio #9200-09 Clone HP6025 10 ul	**Tim-3** R&D #FAB2385P Clone 344823 5 ul	**hIgG4** Southern Bio #9200-09 Clone HP6025 10 ul	**Rat IgG2a** BD #553930 Clone R35-95 5 ul
APC	**NKG2D** Biolegend #320808 Clone 1D11 5 ul	**LAG-3** R&D #FAB2319A Polyclonal 10 ul	**PD-L1** BD #563741 Clone MIH1 5 ul	**PD-L1** BD #563741 Clone MIH1 5 ul	**Perforin** Biolegend #308110 Clone DG9 2 ul	**Goat IgG** R&D #IC108A Polyclonal 10 ul	**Ms IgG2b** Biolegend #400330 Clone MPC-11 2 ul
PerCP/Cy5.5	**CD69** Biolegend #310926 Clone FN50 5 ul	**CD127** eBioScience #45-1278-42 Clone eBioRDR5 5 ul	**CD19** eBioscience #45-0199-42 Clone HIB19 5 ul	**CD66b** Biolegend #305108 Clone G10F5 5 ul	**CD45** eBioscience #45-9459-42 Clone 2D1 5 ul	**Ms IgG1** Biolegend #400150 Clone MOPC-21 5 ul	**CD45** eBioscience #45-9459-42 Clone 2D1 5 ul
AF 700	**CD4** Biolegend #317426 Clone OKT4 5 ul	**CD4** Biolegend #317426 Clone OKT4 5 ul	**CD4** Biolegend #317426 Clone OKT4 5 ul	**CD16** Biolegend #302026 Clone 3G8 5 ul	**CD4** Biolegend #317426 Clone OKT4 5 ul	**CD16** Biolegend #302026 Clone 3G8 5 ul	**CD4** Biolegend #317426 Clone OKT4 5 ul
Pacific Blue	**CD8** Biolegend #300928 Clone HIT8a 5 ul	**CD8** Biolegend #300928 Clone HIT8a 5 ul	**CD8** Biolegend #300928Clone HIT8a 5 ul	**CD11c** BD #562561 Clone B-ly6 5 ul	**CD8** Biolegend #300928 Clone HIT8a 5 ul	**KIR3DL1** Biolegend #312714 Clone DX9 5 ul	**CD8** Biolegend #300928 Clone HIT8a 5 ul
Cy7/PE	**CD45RA** eBioscience #25-0458-42 Clone HI100 5 ul		**NKp30** eBioscience #25-3379-41 Clone AF29-4D12 5 ul	**HLA-DR** Biolegend #307616 Clone L243 5 ul			
BUV395	**CD56** BD #563554 Clone NCAM 16.2 5 ul	**CD56** BD #563554 Clone NCAM 16.2 5 ul	**CD56** BD #563554 Clone NCAM 16.2 5 ul	**CD3 + 56 + 19** Clones NCAM16.2, SJ25C1, and UHCT1 5 ul each	**CD56** BD #563554 Clone NCAM 16.2 5 ul	**CD56** BD #563554 Clone NCAM 16.2 5 ul	**CD56** BD #563554 Clone NCAM 16.2 5 ul
BUV510	**CD3** BD #563109 Clone UHCT1 5 ul	**CD3** BD #565511 Clone UHCT1 5 ul	**CD3** BD #565511 Clone UHCT1 5 ul		**CD3** BD #565511 Clone UHCT1 5 ul	**CD3** BD #565511 Clone UHCT1 5 ul	**CD3** BD #565511 Clone UHCT1 5 ul
APC-H7	**CD45** BD #560178 Clone SK7 5 ul	**CD45** BD #560178 Clone SK7 5 ul	**CD45** BD #560178 Clone SK7 5 ul	**CD45** BD #560178 Clone SK7 5 ul		**CD45** BD #560178 Clone SK7 5 ul	
BUV805				**CD14** BD #565779 Clone M5E2 5 ul			
PerCP	**Propidium Iodide** 50 ng/ml	**Propidium Iodide** 50 ng/ml	**Propidium Iodide** 50 ng/ml	**Propidium Iodide** 50 ng/ml		**Propidium Iodide** 50 ng/ml	

Validation Links

CD3 – Clone UHCT1
https://www.bdbiosciences.com/content/bdb/paths/generate-tds-document.us.748569.pdf

CD4 - Clone OKT4
https://www.biolegend.com/en-us/products/alexa-fluor-700-anti-human-cd4-antibody-3661?pdf=true&displayInline=true&leftRightMargin=15&topBottomMargin=15&filename=Alexa%20Fluor%C2%AE%20700%20anti-human%20CD4%20Antibody.pdf&v=20230114013553

CD8 – Clone HIT8a
https://www.biolegend.com/en-us/products/pacific-blue-anti-human-cd8a-antibody-6659?pdf=true&displayInline=true&leftRightMargin=15&topBottomMargin=15&filename=Pacific%20Blue™%20anti-human%20CD8a%20Antibody.pdf&v=20221026102119

CD11c – Clone B-ly6
https://www.bdbiosciences.com/content/bdb/paths/generate-tds-document.us.562561.pdf

CD14 – Clone M5E2
https://www.bdbiosciences.com/content/dam/bdb/products/global/reagents/flow-cytometry-reagents/research-reagents/single-color-antibodies-ruo/612902_base/pdf/612902.pdf

CD16 – Clone 3G8
https://www.biolegend.com/en-us/products/alexa-fluor-700-anti-human-cd16-antibody-3398?pdf=true&displayInline=true&leftRightMargin=15&topBottomMargin=15&filename=Alexa%20Fluor%C2%AE%20700%20anti-human%20CD16%20Antibody.pdf&v=20230114013553

CD19 - Clone HIB19
https://www.thermofisher.com/order/genome-database/dataSheetPdf?producttype=antibody&productsubtype=antibody_primary&productId=45-0199-42&version=302

CD25 - Clone M-A251
https://www.bdbiosciences.com/content/bdb/paths/generate-tds-document.us.555431.pdf

CD45 – Clone SK7
https://www.bdbiosciences.com/content/bdb/paths/generate-tds-document.us.560178.pdf

CD45 - Clone 2D1
https://www.thermofisher.com/order/genome-database/dataSheetPdf?producttype=antibody&productsubtype=antibody_primary&productId=45-9459-42&version=302

CD45RA – Clone HI100
https://www.thermofisher.com/order/genome-database/dataSheetPdf?producttype=antibody&productsubtype=antibody_primary&productId=25-0458-42&version=302

CD56 – Clone NCAM16.2
https://www.bdbiosciences.com/content/dam/bdb/products/global/reagents/flow-cytometry-reagents/research-reagents/single-color-antibodies-ruo/563554_base/pdf/563554.pdf

CD62L - Clone DREG-56
https://www.biolegend.com/en-us/products/pe-anti-human-cd62l-antibody-653?pdf=true&displayInline=true&leftRightMargin=15&topBottomMargin=15&filename=PE%20anti-human%20CD62L%20Antibody.pdf&v=20230114013553

CD66b - Clone G10F5
https://www.biolegend.com/en-us/products/percp-cyanine5-5-anti-human-cd66b-antibody-6585?pdf=true&displayInline=true&leftRightMargin=15&topBottomMargin=15&filename=PerCP/Cyanine5.5%20anti-human%20CD66b%20Antibody.pdf&v=20220826123057

CD69 - Clone FN50
https://www.biolegend.com/en-us/products/percp-cyanine5-5-anti-human-cd69-antibody-5606?pdf=true&displayInline=true&leftRightMargin=15&topBottomMargin=15&filename=PerCP/Cyanine5.5%20anti-human%20CD69%20Antibody.pdf&v=20230114013553

CD127 - Clone eBioRDR5
https://www.thermofisher.com/order/genome-database/dataSheetPdf?producttype=antibody&productsubtype=antibody_primary&productId=45-1278-42&version=302

DNAM-1 - Clone TX25
https://www.biolegend.com/en-us/products/fitc-anti-human-cd226-dnam-1-antibody-5400?pdf=true&displayInline=true&leftRightMargin=15&topBottomMargin=15&filename=FITC%20anti-human%20CD226%20(DNAM-1)%20Antibody.pdf&v=20230114013553

Granzyme K - Clone GM6C3
https://datasheets.scbt.com/sc-56125.pdf

HLA-DR – Clone L243
https://www.biolegend.com/en-us/products/pe-cyanine7-anti-human-hla-dr-antibody-2862?pdf=true&displayInline=true&leftRightMargin=15&topBottomMargin=15&filename=PE/Cyanine7%20anti-human%20HLA-DR%20Antibody.pdf&v=20230114013553

IL-15RA - Clone eBioJM7
https://www.thermofisher.com/order/genome-database/dataSheetPdf?producttype=antibody&productsubtype=antibody_primary&productId=11-7159-42&version=302

KIR3DL1 (CD158e1) – Clone DX9
https://www.biolegend.com/en-us/products/brilliant-violet-421-anti-human-cd158e1-kir3dl1-nkb1-antibody-7296?pdf=true&displayInline=true&leftRightMargin=15&topBottomMargin=15&filename=Brilliant%20Violet%20421™%20anti-human%20CD158e1%20(KIR3DL1,%20NKB1)%20Antibody.pdf&v=20230114013553

LAG-3 – Polyclonal
https://resources.rndsystems.com/pdfs/datasheets/fab2319a.pdf?v=20230505

NKG2D - Clone 1D11
https://www.biolegend.com/en-us/products/apc-anti-human-cd314-nkg2d-antibody-3017?pdf=true&displayInline=true&leftRightMargin=15&topBottomMargin=15&filename=APC%20anti-human%20CD314%20(NKG2D)%20Antibody.pdf&v=20221115073101

NKp30 – Clone AF29-4D12
https://www.thermofisher.com/order/genome-database/dataSheetPdf?producttype=antibody&productsubtype=antibody_primary&productId=25-3379-41&version=302

NKp44 - Clone P44-8
https://www.biolegend.com/en-us/products/pe-anti-human-cd336-nkp44-antibody-3849?pdf=true&displayInline=true&leftRightMargin=15&topBottomMargin=15&filename=PE%20anti-human%20CD336%20(NKp44)%20Antibody.pdf&v=20230114013553

Nkp80 - Clone 5D12
https://www.biolegend.com/en-us/products/ultra-leaf-purified-anti-human-nkp80-antibody-19179?pdf=true&displayInline=true&leftRightMargin=15&topBottomMargin=15&filename=Ultra-LEAF%E2%84%A2%20Purified%20anti-human%20NKp80%20Antibody.pdf&v=20230114013553

PD-L1 - Clone M1H1
https://www.bdbiosciences.com/content/bdb/paths/generate-tds-document.us.563741.pdf

Perforin - Clone DG9
https://www.biolegend.com/en-us/products/alexa-fluor-647-anti-human-perforin-antibody-3156?pdf=true&displayInline=true&leftRightMargin=15&topBottomMargin=15&filename=Alexa%20Fluor%C2%AE%20647%20anti-human%20Perforin%20Antibody.pdf&v=20230114043032

TIM-3 - Clone 344823
https://www.rndsystems.com/products/human-tim-3-pe-conjugated-antibody-344823_fab2365p#ds_datasheets

Secondary antibodies

Goat IgG – polyclonal
https://resources.rndsystems.com/pdfs/datasheets/ic108a.pdf?v=20230505&_ga=2.12993783.1700577917.1683318599-1221737743.1683318598

Human IgG4 - Clone HP6025
https://www.southernbiotech.com/wp/techbul/index.php/9200-09

Mouse IgG1k - Clone MOPC-21
https://www.biolegend.com/en-us/products/percp-cyanine5-5-mouse-igg1-kappa-isotype-ctrl-4205?pdf=true&displayInline=true&leftRightMargin=15&topBottomMargin=15&filename=PerCP/Cyanine5.5%20Mouse%20IgG1,%20%CE%BA%20Isotype%20Ctrl%20Antibody.pdf&v=20230114043032

Mouse IgG2b - Clone MPC-11

https://www.biolegend.com/en-us/products/alexa-fluor-647-mouse-igg2b-kappa-isotype-ctrl-2691?pdf=true&displayInline=true&leftRightMargin=15&topBottomMargin=15&filename=Alexa%20Fluor%C2%AE%20647%20Mouse%20IgG2b,%20%CE%BA%20Isotype%20Ctrl%20Antibody.pdf&v=20230114043032

Rat IgG2b - Clone RTK4530
https://www.biolegend.com/en-us/products/fitc-rat-igg2b-kappa-isotype-ctrl-1854?pdf=true&displayInline=true&leftRightMargin=15&topBottomMargin=15&filename=FITC%20Rat%20IgG2b,%20%CE%BA%20Isotype%20Ctrl%20Antibody.pdf&v=20230114013553

Rat IgG2a - Clone R35-95
https://www.bdbiosciences.com/content/bdb/paths/generate-tds-document.us.553930.pdf

Supplementary Table 5: Change in PD-L1 expression from Baseline

Table of immunohistochemistry (IHC) of PD-L1 expression for each patient on trial. PD-L1 expression utilized VENTANA PD-L1 (SP263) assay and was calculated using the tumor proportion score (TPS) with >1% considered the cut-off. Analysis was done by a singular study pathologist. Individual patients are shown with baseline and on-treatment expression findings as well as whether a changes was documented after treatment. Patients are stratified by dose level.

Seq #	Diagnosis	PD-L1 Expression: Baseline	PD-L1 Expression: Post-Induction	Change
028	Renal Cell Carcinoma	<1%	<1%	↔
033	Breast	>1%	>1%	↔
036	Renal Cell Carcinoma	>1%	>1%	↔
037	Ovarian	>1%	>1%	↔
038	Mesothelioma	>1%	<1%	↓
001	Breast	>1%	>1%	↔
002	Renal Cell Carcinoma	<1%	>1%	↑
003	Esophageal (adeno)	>1%	>1%	↔
004	Ovarian	<1%	>1%	↑
005	Papillary Renal Cell Carcinoma	>1%	<1%	↓
006	Esophageal (squamous)	<1%	<1%	↔
007	Breast	<1%	<1%	↔
008	Endometrial	<1%	<1%	↔
009	Ovarian	<1%	<1%	↔
012	Breast	<1%	>1%	↑
013	Lung (Squamous)	>1%	>1%	↔
014	Endometrial	<1%	<1%	↔
016	Ovarian	<1%	>1%	↑
019	Gastric	>1%	<1%	↓
022	Anal	>1%	<1%	↓
023	Urothelial	>1%	>1%	↔

25 mcg/m^2
50 mcg/m^2
75 mcg/m^2
100 mcg/m^2

Supplementary Table 6: Change in CD68 expression from Baseline

Table of IHC of CD68 expression for each patient on trial. Expression was performed according to local protocols on an automated immunostainer and calculated as positively staining cells per high powered field (hpf). Analysis was done by a singular study pathologist. Individual patients are shown with baseline and on-treatment expression findings as well as whether a change was documented after treatment. Patients are stratified by dose level.

Seq #	Diagnosis	CD-68 Expression Baseline (cells/hpf)	CD-68 Expression Post-Induction (cells/hpf)	Change
028	Renal Cell Carcinoma	5	15	↑
033	Breast	25	50	↑
036	Renal Cell Carcinoma	30	50	↑
037	Ovarian	100	25	↓
038	Mesothelioma	20	4	↓
001	Breast	70	70	↔
002	Renal Cell Carcinoma	10	>100	↑
003	Esophageal (adeno)	15	5	↓
004	Ovarian	100	10	↓
005	Papillary Renal Cell Carcinoma	50	1	↓
006	Esophageal (squamous)	>100	5	↓
007	Breast	15	40	↑
008	Endometrial	5	50	↑
009	Ovarian	10	10	↔
012	Breast	10	50	↑
013	Lung (Squamous)	5	25	↑
014	Endometrial	50	60	↑
016	Ovarian	20	100	↑
019	Gastric	20	35	↑
022	Anal	n/a	n/a	n/a
023	Urothelial	25	30	↑

25 mcg/m^2

50 mcg/m^2

75 mcg/m^2

100 mcg/m^2

GU-084 Combination immunotherapy with interferon-gamma and nivolumab for patients with advanced solid tumors: A Phase 1 Study

Supported by Horizon Pharma, LLC

Principal Investigator:
Matthew Zibelman, MD
Fox Chase Cancer Center
333 Cottman Avenue
Philadelphia PA 19111
Tel: 215-728-3889
Fax: 215-728-3639
matthew.zibelman@fccc.edu

Co-Investigator:
Elizabeth R. Plimack, MD, MS
Fox Chase Cancer Center
333 Cottman Avenue
Philadelphia, PA 19111
Phone 215-728-3889
elizabeth.plimack@fccc.edu

Sub-Investigators:
Crystal Denlinger, MD, FACP
Fox Chase Cancer Center
333 Cottman Avenue
Philadelphia, PA 19111
Phone 215-728-5673
crystal.denlinger@fccc.edu

Efrat Dotan, MD
Fox Chase Cancer Center
333 Cottman Avenue
Philadelphia, PA 19111
Phone 215-728-5673
efrat.dotan@fccc.edu

Anthony Olszanski, RPh, MD
Fox Chase Cancer Center
333 Cottman Avenue
Philadelphia, PA 19111
Phone 215-728-5673
anthony.olszanski@fccc.edu

Copyright© 2016 Fox Chase Cancer Center® Office of Clinical Research. All rights reserved.

Daniel M. Geynisman, MD
Fox Chase Cancer Center
333 Cottman Avenue
Philadelphia, PA 19111
Phone 215-728-3889
daniel.geynisman@fccc.edu

Namrata Vijayvergia, MD
Fox Chase Cancer Center
333 Cottman Avenue
Philadelphia, PA 19111
Phone 215-728-5673
namrata.vijayvergia@fccc.edu

Rohit Walia, MD,
Fox Chase Cancer Center
333 Cottman Avenue
Philadelphia, PA 19111
Phone 215-728-3024
Rohit.Walia@fccc.edu

Michael Hall, MD, MS
Fox Chase Cancer Center
333 Cottman Avenue
Philadelphia, PA 19111
Phone 215-728-2861
Michael.hall@fccc.edu

Igor Astsaturov, MD, PhD
Fox Chase Cancer Center
333 Cottman Avenue
Philadelphia, PA 19111
Phone 215-214-4297
Igor.astsaturov@fccc.edu

Jonathan Cheng, MD
Fox Chase Cancer Center
333 Cottman Avenue
Philadelphia, PA 19111
Phone 215-728-4300
Jonathan.cheng@fccc.edu

Copyright© 2016 Fox Chase Cancer Center® Office of Clinical Research. All rights reserved.

Laboratory Collaborators: Siddharth Balachandran, Ph.D.
Fox Chase Cancer Center
333 Cottman Avenue
Philadelphia, PA 19111
Phone 215-214-1527
siddharth.balachandran@fccc.edu

Kerry S. Campbell, Ph.D.
Fox Chase Cancer Center
333 Cottman Avenue
Philadelphia, PA 19111
Phone 215-728-7761
kerry.campbell@fccc.edu

Edna Cukierman, Ph.D.
Fox Chase Cancer Center
333 Cottman Avenue
Philadelphia, PA 19111
Phone 215-214-4218
edna.cukierman@fccc.edu

Statistician: Karthik Devarajan, PhD
Fox Chase Cancer Center
333 Cottman Avenue
Philadelphia PA 19111
Phone: 215-728-2794
karthik.devarajan@fccc.edu

Initial Version Date: 10/26/2015
Amendment 1 Version Date: 06/06/2016
Amendment 2 Version Date: 02/14/2017

Copyright© 2016 Fox Chase Cancer Center® Office of Clinical Research. All rights reserved.

Study Schema

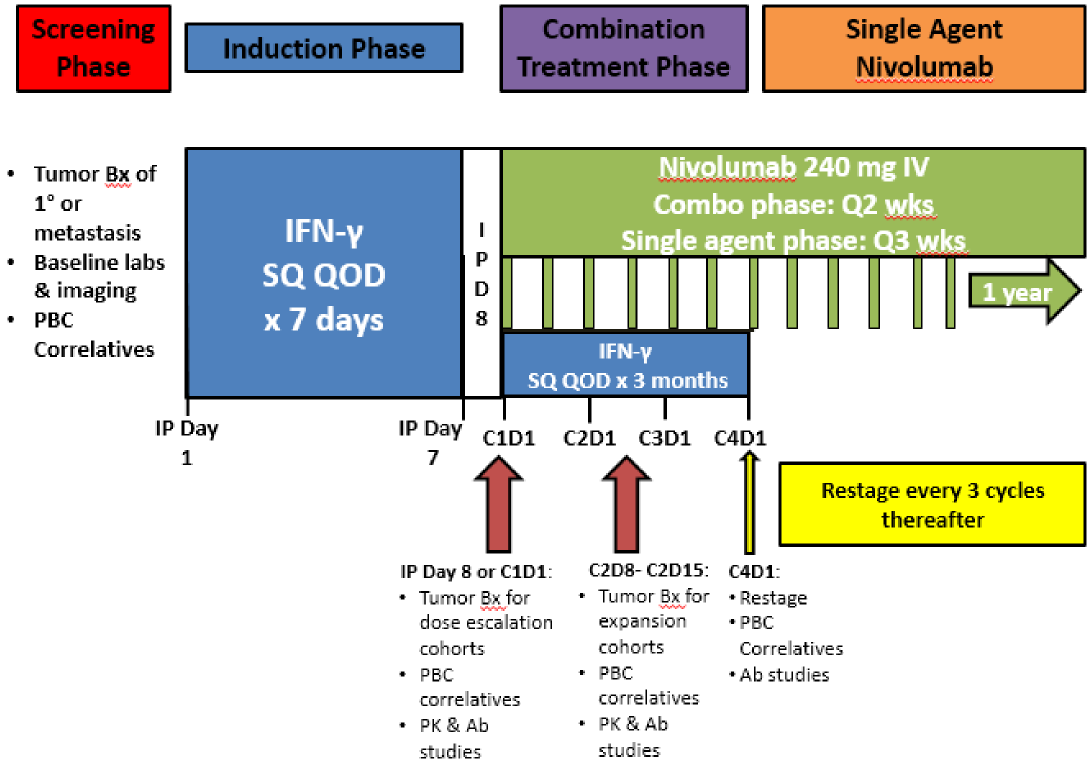

*Note: Patients who enrolled in the dose escalation phase received nivolumab at 3mg/kg.

Copyright© 2016 Fox Chase Cancer Center® Office of Clinical Research. All rights reserved.

Population

- Dose escalation: patients with select solid tumors and progression after at least one prior systemic therapy
- Expansion cohorts in patients with advanced esophagogastric carcinoma and patients who have progressed on prior PD-1 pathway directed therapies

↓

Treatment

- *Interferon-gamma (IFN-γ):* starting dose 50 mcg/m^2 subcutaneously
- *Nivolumab:* 3 mg/kg intravenously (adjusted to 240 mg fixed due to FDA change)

Induction phase: IFN-γ every other day alone for 1 week

Combination phase: IFN-γ every other day & Nivolumab every 2 weeks for 3 months

↓

Disease Assessment

- History and Physical every 2 weeks during combination phase, then every 3 weeks during single agent phase
- Computed Tomography (CT) at conclusion of combination phase, then every 3 cycles during single agent phase

↓

Continue treatment until:

- Disease progression (treatment beyond first progression allowed if clinically appropriate per treating clinician)
- Unacceptable toxicity
- Patient preference

↓

Follow up

Follow every 2-3 months for study related toxicities and survival

Copyright© 2016 Fox Chase Cancer Center® Office of Clinical Research. All rights reserved.

Table of Contents

STUDY SCHEMA 4

TABLE OF CONTENTS 6

1.0 INTRODUCTION 10
1.1. STUDY RATIONALE 10
1.2. AGENTS UNDER INVESTIGATION 12
1.2.1. Interferon-gamma 1b (IFN-γ 1b, Actimmune®) 12
1.2.2. Nivolumab (Opdivo®) 13
1.3. CORRELATIVE TESTING 15
1.3.1. Biopsy Specimen Analysis 15
1.3.2. Peripheral Blood Correlative Analysis 15
1.3.3. Pharmacokinetic (PK) and neutralizing antibody assessments 15

2.0 OBJECTIVES 15
2.1. PRIMARY OBJECTIVE 15
2.2. SECONDARY OBJECTIVES 15
2.3. EXPLORATORY OBJECTIVES 16

3.0 STUDY PLAN OVERVIEW 16
3.1. DESCRIPTION OF STUDY DESIGN, POPULATION AND DURATION OF STUDY THERAPY 16

4.0 PATIENT SELECTION INCLUSION & EXCLUSION CRITERIA 16
4.1. INCLUSION CRITERIA 16
4.2. EXCLUSION CRITERIA 18
4.3. INCLUSION OF WOMEN AND MINORITIES 19
4.4. PREGNANCY 19
4.5. PATIENT REGISTRATION 20

5.0 TREATMENT PLAN 20
5.1. GENERAL OVERVIEW 20
5.2. BASELINE ASSESSMENTS 20
5.2.1. History and Physical 20
5.2.2. Imaging 20
5.2.3. Laboratory studies 21
5.3. CORRELATIVE STUDIES 21
5.3.1. Tumor Biopsy 21
5.3.2. Serum Correlatives 21
5.4. TREATMENT PHASES 21
5.4.1. Induction Phase 21
5.4.2. Combination Phase 22

Copyright© 2016 Fox Chase Cancer Center® Office of Clinical Research. All rights reserved.

5.4.3. *Single Agent Phase* *23*
5.5. TREATMENT ADMINISTRATION 23
5.5.1. *Prophylactic or Supportive Medications* *24*
5.6. DOSE ESCALATION COHORT 24
5.7. EXPANSION COHORTS 26
5.8. RECOMMENDED PHASE 2 DOSE DEFINITION 27
5.9. CONCOMITANT MEDICATIONS, SUPPORTIVE CARE, EXCLUDED THERAPIES AND RESTRICTIONS 27
5.9.1. *Supportive Care* *27*
5.9.2. *Concomitant Medications and Excluded Therapies* *28*
5.10. DURATION OF THERAPY 30
5.11. DURATION OF FOLLOW UP 30
5.12. TREATMENT BEYOND DISEASE PROGRESSION 30
5.13. CRITERIA FOR DISCONTINUATION 31
5.13.1. *General Reasons for Discontinuation or Withdraw from Study* *31*
5.13.2. *Discontinuation Criteria from IFN-γ (IP only)* *31*
5.13.3. *Discontinuation Criteria from Nivolumab (CP or single-agent phase)* *31*

6.0 DOSE MODIFICATIONS 32
6.1. GENERAL PRINCIPLES 32
6.2. DOSE LEVEL ADJUSTMENT – 33
6.3. TOXICITY ASSESSMENT AND GRADING 33
6.4. DEFINITION OF DLTS 33
6.5. INSTRUCTIONS FOR SPECIFIC DOSE MODIFICATIONS 34
6.5.1. *Treatment of Drug-Related Infusion Reactions* *37*

7.0 STUDY DRUGS 38
7.1. IFN-Γ FORMULATION, PRODUCT IDENTIFICATION, PACKAGE AND LABELING 38
7.1.1. *Product description* *38*
7.1.2. *Availability* *39*
7.1.3. *Solution preparation* *39*
7.1.4. *Storage requirements and Stability* *39*
7.1.5. *Route of administration* *39*
7.2. NIVOLUMAB 39
7.2.1. *Product Description* *39*
7.2.2. *Availability* *39*
7.2.3. *Solution preparation* *39*
7.2.4. *Storage Requirements and Stability* *39*
7.2.5. *Route of Administration* *39*
7.3. DRUG ORDERING, STORAGE AND HANDLING 39
7.4. DESTRUCTION OF DRUG 40
7.5. RECORDS TO BE KEPT AT SITE; DISPENSING AND ACCOUNTABILITY 40

Copyright© 2016 Fox Chase Cancer Center® Office of Clinical Research. All rights reserved.

8.0 CORRELATIVE /SPECIAL STUDIES 40
8.1. BIOPSY SPECIMEN ANALYSIS 40
8.2. PERIPHERAL BLOOD ANALYSIS 41
8.3. PK AND NEUTRALIZING ANTIBODY ASSESSMENTS 42

9.0 STUDY CALENDAR 43

10.0 ADVERSE EVENTS 45
10.1. DEFINITIONS 45
10.2. SEVERITY RATING 45
10.3. ATTRIBUTION/RELATIONSHIP TO STUDY DRUG 45
10.4. EXPECTEDNESS 45
10.5. RECORDING AND REPORTING RESPONSIBILITIES 46
10.5.1. Investigative site recording responsibilities: 46
10.5.2. Investigative site reporting responsibilities: 46
10.5.3. CTO Reporting Responsibilities: 47
10.5.4. SAE Reporting to Horizon 48
10.6. PREGNANCY 48

11.0 MEASURES OF EFFECT 48
11.1. RESPONSE EVALUATION CRITERIA IN SOLID TUMORS (RECIST) 48
11.2. DEFINITIONS 49
11.3. DISEASE PARAMETERS 49
11.4. METHODS FOR EVALUATION OF MEASURABLE DISEASE 50
11.5. RESPONSE CRITERIA 52
11.5.1. Evaluation of Target Lesions 52
11.5.2. Evaluation of Non-Target Lesions 53
11.5.3. Evaluation of Best Overall Response (BOR) 53
11.6. DURATION 54
11.7. PROGRESSION-FREE SURVIVAL 54

12.0 STATISTICAL CONSIDERATIONS 54
12.1. STUDY DESIGN/ENDPOINTS 54
12.1.1. Primary Endpoint: Safety and Tolerability 54
12.1.2. Secondary Endpoint: ORR in the individual disease specific dose expansion cohorts 55
12.1.3. Secondary Endpoints: PFS and OS in the individual disease specific dose expansion cohorts 56
12.1.4. Secondary Endpoint: OS at 1 year in the disease specific dose-expansion cohorts 56
12.1.5. Analysis of Correlative Endpoints 56
12.2. SAMPLE SIZE/ACCRUAL RATE 56
12.2.1. Planned Sample Size 56
12.3. REPORTING AND EXCLUSIONS 57

Copyright© 2016 Fox Chase Cancer Center® Office of Clinical Research. All rights reserved.

13.0 DATA AND SAFETY MONITORING PLAN..58
13.1. MONITORING PLAN 58
13.2. DATA SAFETY MONITORING BOARD 58
14.0 ADMINISTRATIVE..58
14.1. DATA REPORTING 58
14.2. RETENTION OF RECORDS 59
14.3. STUDY AGENTS..59
14.4. INFORMED CONSENT 59
15.0 REFERENCES 60

Copyright© 2016 Fox Chase Cancer Center® Office of Clinical Research. All rights reserved.

1.0 Introduction

1.1. Study Rationale

Immunotherapy is an evolving treatment paradigm for malignancy, with a variety of strategies currently being employed in the clinic and in on-going trials. Specifically, novel immune checkpoint inhibitors have shown promising and durable clinical efficacy in various tumor types, heralded by drugs targeting the programmed cell death 1 (PD-1) pathway. The PD-1/PD-L1 pathway regulates host immune surveillance via co-inhibitory receptors expressed on T-cells and enable tumors to evade immune detection and diminish the anti-tumor response.[10] By inhibiting these "checkpoint" receptors or their associated ligands (programmed cell death ligand 1 and 2, PD-L1 and PD-L2, respectively), T-cell activation and subsequent immune proliferation can proceed unabated, increasing the likelihood of a durable, immune-mediated anti-tumor response.[9] As proof of concept, several PD-1/PD-L1 checkpoint inhibitors have produced deep and durable responses in a variety of tumor types, including genitourinary malignancies such as renal cell carcinoma (RCC) and urothelial cancer (UC).[11-14, 16, 24, 25, 36] Specifically three agents targeting the PD-1 pathway have garnered approval for the treatment of patients with refractory metastatic cancers across 6 malignancies. The PD-1 inhibitors nivolumab and pembrolizumab have both been approved for treating certain patients with metastatic melanoma, non-small cell lung cancer (NSCLC), and squamous cell cancer of the head and neck (SCCHN), while nivolumab has also been approved for RCC, UC and refractory Hodgkin's lymphoma. All of these approvals were based on level one evidence from randomized trials demonstrating improved survival compared to standard of care therapy.[33, 34, 37-43] Additionally, the PD-L1 inhibitor atezolizumab was approved for the treatment of platinum-resistant metastatic UC (mUC) based on evidence from a single arm study, and it was approved for patients with metastatic NSCLC based on evidence from 2 randomized trials.[44-46] Response rates of single agent PD-1 and PD-L1 inhibitors in previously treated patients with a variety of tumor types in these early studies have ranged from 15-38%, so while these drugs have marked improvements over the prior standards of care, clearly more work can be done to increase the percent of patients that benefit.

Initial studies suggested that baseline PD-L1 tumor cell expression could serve as a key biomarker for response to PD-1 pathway inhibition, but subsequent investigation has determined that the relationship is more complex than initially suspected.[14, 50] Some explanations for this discordance may include intratumoral heterogeneity affecting the PD-L1 expression observed at various biopsy sites, differences and lack of standardization in available assays for PD-L1 testing across trials, and the dynamic nature of the tumor-immune cell interaction causing fluctuations in expression levels.[50-52] There also has been uncertainty as to whether PD-L1 expression matters more in the tumor cells themselves, or in tumor infiltrating lymphocytes (TILs) that invade the tumor microenvironment. Tumor PD-L1 up-regulation has been the factor most strongly correlated with response to PD-1 blockade, but clearly other factors are involved in determining which patients will ultimately benefit.[53] Alternatively, given

Copyright© 2016 Fox Chase Cancer Center® Office of Clinical Research. All rights reserved.

that many of the early studies of PD-1 inhibition analyzed archival instead of fresh tumor biopsies, one possibility may be that the level of PD-L1 expression at the time of PD-1 blockade is integral to optimizing response. Thus, this begs the question: if PD-L1 expression in the tumor microenvironment could be reliably up-regulated, could the overall response rate (ORR) of PD-1 inhibition be improved?

Interferon-gamma (IFN-γ) is a commercially available type II interferon that has been previously studied in a variety of solid tumors, but is currently only FDA-approved for the treatment of patients with chronic granulomatous disease (CGD) and severe malignant osteopetrosis (SMO).[27] However, IFN-γ is a key regulator of PD-L1 expression. Preclinical studies using cell lines have demonstrated that IFN-γ enhances cellular PD-L1 expression on host endothelial cells and on some tumor cells.[54-56] Compared to the type I interferons (IFN-α and IFN-β), IFN-γ has exhibited superior and more durable enhancement of PD-L1 expression in endothelial cell lines.[55] Taube and colleagues showed that 98% of analyzed melanoma tumors that stained PD-L1 positive were also associated with TILs, while TILs were seen in only 28% of PD-L1 negative tumors. In the PD-L1 positive tumors, IFN-γ was shown to be present at the interface between TILs and adjacent melanoma cells, while PD-L1 negative melanomas demonstrated an absence of IFN-γ, suggesting IFN-γ is essential for PD-L1 up-regulation in the tumor microenvironment.[57] The effect of exogenous IFN-γ given in combination with PD-L1 blockade on anti-tumor cytotoxicity has also been evaluated in the preclinical setting. Mimura and colleagues treated various tumor cells lines with IFN-γ, all of which exhibited consistent up-regulation of PD-L1 expression, but the IFN-γ also resulted in impaired anti-tumor cytotoxicity. However, when the cell lines were treated with IFN-γ in the presence of an anti-PD-L1 monoclonal antibody, not only was baseline anti-tumor cytotoxicity restored, but it exceeded levels in untreated controls.[58] These studies may explain why IFN-γ was ineffective as a single agent in the treatment of cancer patients, but provides ample evidence of the potential of a combination strategy in a clinical setting.

Based on this strong pre-clinical evidence, we theorize that IFN-γ will increase PD-L1 expression by tumor cells and boost the cytotoxic potential of immune cells, resulting in an improved ORR with PD-1 inhibition. While previous phase III studies of single agent IFN-γ in patients with melanoma and RCC did not improve outcomes, this was prior to the knowledge of the role of the PD-1 pathway in tumor surveillance, which may have unwittingly hampered the drug's effectiveness.[28, 29] In fact, in a study performed at Fox Chase Cancer Center (FCCC) by Dr. Plimack, Dr. Campbell and colleagues in patients with RCC, PD-1 expression on peripheral blood mononuclear cells (PBMCs) correlated with disease expression, and their conclusion suggested that combining PD-1 inhibitors with various cytokine therapies may potentiate natural killer cells and other immune effectors to mount a more effective anti-tumor response.[59] Therefore, we propose a phase I dose escalation/dose expansion clinical trial of combined immunotherapy with nivolumab and IFN-γ in patients with advanced solid tumors who have demonstrated progression of disease on at least one prior systemic therapy in the metastatic setting. The goals of the trial would be to investigate the safety and tolerability of the combination, as well as assess for signals of increased

Copyright© 2016 Fox Chase Cancer Center® Office of Clinical Research. All rights reserved.

ORR as compared to prior single agent PD-1 pathway blockade trials. The initial cohort would include patients with tumors that have clear evidence in the clinical or preclinical setting of PD-L1 expression, as those would be the most likely to benefit from IFN-γ priming.

Once safety is established in the initial dose finding cohort, we plan to expand into two separate cohorts. One cohort will enroll patients with advanced solid tumors where PD-1 pathway drugs have become the standard of care but where patients have experienced clinical progression on one of these agents. As of January 2017 this includes melanoma, RCC, UC, NSCLC, and SCCHN, but as approvals expand over time, new patients may become eligible. Patients in this cohort will have to have progressed on treatment with a prior PD-1 pathway inhibitor without toxicity that would prohibit further administration. One theory of this study is that combination therapy may overcome single agent resistance by optimizing the immunogenic milieu, thus including patients with primary or secondary anti-PD-1 resistance, a patient population with limited clinical trial options, is reasonable. The other cohort would include patients with esophagogastric carcinomas, a mixed disease group without access to standard PD-1 pathway inhibition currently, but where some clinical evidence exists.[60-62] In the dose escalation portion of this study, the first 2 esophagogastric carcinoma patients both experienced clinical disease stabilization for > 8 months, warranting further study in this population

The design of the trial takes into account the proposed hypothesis, namely that IFN-γ will be used to induce PD-L1 up-regulation in tumor cells to increase the likelihood of response to PD-1 inhibition with nivolumab. Thus, we will start all patients on an induction phase of IFN-γ alone, initially at 50 mcg/m^2, every other day for one week, which is the FDA-approved dose and the dose at which evidence of immunogenic activity has been previously described.[31] Patients will then enter a combination phase, at which time they will receive both drugs simultaneously at the FDA-approved dose and schedule and be monitored for dose limiting toxicities (DLTs) for six weeks. After 3 months of combination dosing, all patients still on study will stop IFN-γ and continue on single-agent nivolumab every three weeks for up to one year. The change to every three week dosing is based on data in patients with RCC that the dose interval with nivolumab does not adversely influence efficacy.[16] Importantly, this study will incorporate pre-treatment and on treatment biopsies and serum correlates for all patients in order to evaluate the effect of IFN-γ and ascertain proof-of-concept that it will lead to increased PD-L1 expression. This concept aims to improve the ORR of PD-1 inhibition in refractory, advanced cancer patients, while simultaneously expanding the knowledge base of the interaction between such inhibitors, cytokines, and the tumor microenvironment.

1.2. Agents under Investigation

1.2.1. Interferon-gamma 1b (IFN-γ 1b, Actimmune®)

IFN-γ is a cytokine and is the only type II interferon. It is naturally produced mainly by natural killer cells and T-lymphocytes and works as an

Copyright© 2016 Fox Chase Cancer Center® Office of Clinical Research. All rights reserved.

immunomodulator in various capacities. In its role as an immunomodulator it is 100-10,000 times more potent than the type I interferons. IFN-γ has many functions physiologically, including but not limited to regulating major histocompatibility complex (MHC) expression, activating the differentiation and function of phagocytes, augments interactions between macrophages and T-cells, and plays a key role in regulating T-cell subsets to determine the type of immune effector function during a specific immune response.[27] However, for our purposes, the most important function that we are hoping to capitalize on would be its proclivity to up-regulate PD-L1 expression.

Recombinant IFN-γ 1b (Actimmune®) is commercially available and approved for the treatment of the rare pediatric conditions chronic granulomatous disease (CGD) and severe malignant osteoporosis (SMO).[27] It has, however, previously been evaluated in patients with advanced malignancies and thus has a known and proven safety and toxicity profile. Previous phase III trials of IFN-γ as a single agent in advanced RCC and melanoma did not improve outcomes compared to their respective comparator arms, but it was safe and reasonably well tolerated.[28, 29] Additionally, Devane and colleagues performed a dose titration study in healthy volunteers to establish the best tolerated dosing and schedule in adults in regards to the onset of flu-like symptoms.[30]

IFN-γ will be self-administered as a subcutaneous (SQ) injection at a starting dose of 50 mcg/m^2 on an every other day basis. This starting dose was selected based on data from a phase I dose-finding study of patients with resected melanomas performed by Maluish and colleagues which demonstrated evidence of enhanced immunologic activity (as determined by repeated measurements of hydrogen peroxide levels from monocytes and natural killer cell activity) at various doses and routes of administration.[31] Based on their results and conclusions, IFN-γ dosing from 10-100 mcg/m^2 achieved consistently high immunologic effects with a tolerable side effect profile. The 50 mcg/m^2 is the approved dose in CGD and SMO and was deemed the appropriate starting dose level. Data from the Maluish study also suggested that administration SQ every other day may be the optimal dose to maintain immunologic pressure.

The recombinant form of IFN-γ is rapidly cleared after intravenous administration, but is absorbed more slowly by the SQ route.[32] Following SQ injection, greater than 89% of the dose given is absorbed. As measured at a 100 mcg/m^2 dose, the mean elimination half-life is 5.9 hours. Peak plasma concentration is achieved at seven hours following SQ dosing. No drug accumulation has been reported after twelve consecutive daily injections using a 100 mcg/m^2 dose. While studies in healthy volunteers have failed to document detection of IFN-γ in the urine of healthy subjects, animal studies have suggested some urinary excretion may be present, but predominant elimination is via the liver.

1.2.2. **Nivolumab (Opdivo®)**

Copyright© 2016 Fox Chase Cancer Center® Office of Clinical Research. All rights reserved.

Nivolumab is a monoclonal antibody (mAb) that targets the PD-1 receptor on immune cells. It is produced and marketed by Bristol-Myers Squibb and as of January 2017 has been USFDA-approved for use in patients with advanced metastatic melanoma, renal cell carcinoma (RCC), non-small cell lung cancer (NSCLC), squamous cell cancers of the head and neck (SQCHN), urothelial carcinoma (UC) and refractory Hodgkin's lymphoma. Nivolumab is a fully human IgG4 mAb that does not activate antibody dependent cellular cytotoxicity (ADCC) but is believed to bind PD-1 to prevent interaction with its ligands, most specifically PD-L1, thereby preventing innate mechanisms of immune inhibition and permitting a more robust anti-tumor response.

Initial phase 1 studies explored nivolumab administration in various refractory solid tumors and demonstrated deep and durable responses in subsets of patients with melanoma, RCC, and NSCLC.[11, 14] The approval of nivolumab for metastatic melanoma was based on initial data from an open-label, phase III trial of nivolumab versus physician's choice of chemotherapy in ipilimumab-refractory disease.[33] The drug exhibited an ORR of 31.7% in the first 120 patients treated, versus only 10.6% in the group receiving chemotherapy. The drug was also better tolerated than chemotherapy, with only 5% of patients experiencing a grade 3-4 serious AE. In squamous NSCLC, expedited approval was based on data from the randomized phase III CheckMate-017 trial, which randomized patients to either nivolumab or docetaxel after failure of first-line platinum-based chemotherapy. The trial was closed early due to achievement of an OS benefit in the investigational arm, exhibiting a 41% reduction in the risk of death in patients who received nivolumab. In patients with metastatic RCC, nivolumab has demonstrated an OS benefit as compared to everolimus in patients refractory to at least one prior tyrosine kinase inhibitor.[34] Data for nivolumab in other tumor types is not reviewed here, but was sufficient for USFDA approval based on level one evidence from randomized trials.

Nivolumab will be administered as a fixed dose of 240 mg intravenously (IV) over one hour on an every two week schedule during the combination phase with IFN-gamma, but will be adjusted to every three weeks during the single agent phase. No dose reductions of nivolumab will be permitted, but holding doses for recovery from acute toxicity and disease-related occurrences is acceptable. The every two-week dosing schedule during the combination phase follows the FDA-approval for this agent in melanoma and NSCLC and will allow for shorter period of combination therapy. The change to every three week dosing is based on a randomized phase II trial published by Motzer and colleagues evaluating the dose-response relationship of varying doses of nivolumab given every three weeks in patients with metastatic RCC.[16] The results suggested no dose-response relationship existed, though there was a non-significant trend towards greater toxicity at higher doses. The 240 mg dose is the current, FDA-approved dose, as per change in recommendations by the FDA on September 15, 2016.

Copyright© 2016 Fox Chase Cancer Center® Office of Clinical Research. All rights reserved.

Pharmacokinetic (PK) studies of nivolumab have been assessed in over 900 patients across multiple studies.[35] The geometric mean clearance and elimination half-life were found to be 9.5 mL/h and 26.7 days, respectively. When administered at the dose of 3 mg/kg every two weeks, steady-state concentrations of nivolumab were reached by 12 weeks, with approximately a 3-fold systemic accumulation. Total nivolumab exposure increased proportionally over the dose range of 0.1-10 mg/kg. No formal PK drug-drug interaction studies have been performed with nivolumab.

1.3. Correlative Testing

1.3.1. Biopsy Specimen Analysis

Biopsy specimen analysis will endeavor to serve as proof of principle of the central hypothesis that IFN-γ priming will increase PD-L1 expression on tumors. Each patient will undergo two mandatory biopsies during the course of the study. In the dose escalation portion, a second biopsy will be performed after IFN-γ induction but prior to initiation of nivolumab. The principle purpose of this timing will be to directly evaluate whether PD-L1 expression is truly up-regulated after the IFN-γ induction. Biopsies for the patients in the dose expansion portion of the study will be performed during the combination phase. Complete detail on this design is discussed in section 5.0. Furthermore, biopsy specimens will be analyzed utilizing several platforms for correlative analysis, which is further detailed in section 8.0 and in the laboratory manual for this study.

1.3.2. Peripheral Blood Correlative Analysis

Peripheral blood samples will be analyzed for evidence of the immunologic and pharmacodynamic effect of IFN-γ administration in the periphery by assessing known targets of IFN-γ activity, frequencies of T cell subsets (including regulatory T cells), biomarkers of immune activation, and various forms of PD-1/PD-L1 receptors evaluable in the blood.

1.3.3. Pharmacokinetic (PK) and neutralizing antibody assessments

PK and antibody studies of both IFN-γ and nivolumab will be assessed throughout the study as detailed in the study calendar

2.0 Objectives

2.1. Primary Objective

To evaluate the safety and tolerability of the combination of nivolumab and IFN-γ and determine the recommended phase 2 dose (RP2D)

2.2. Secondary Objectives

Copyright© 2016 Fox Chase Cancer Center® Office of Clinical Research. All rights reserved.

1. To evaluate the investigator assessed ORR using standard response evaluation criteria in solid tumors (RECIST) version 1.1 for each expansion cohort separately
2. To evaluate median progression free survival (PFS) for each expansion cohort separately
3. To evaluate median overall survival (OS) for each expansion cohort separately
4. To assess OS at 1 year for patients in the expansion cohorts
5. To investigate the relationship between PD-L1 expression on tumor cells and on immune cells in the tumor microenvironment before and after treatment initiation

2.3. Exploratory Objectives

1. To investigate whether the change in PD-L1 expression in tumor biopsy samples correlates with ORR
2. To assess the effect of IFN-γ treatment on markers of IFN-γ activity at various time points before, during, and after drug administration
3. To evaluate for changes in soluble PD-L1 concentrations and PD-1 expression on circulating immune cells before and during study treatment
4. To explore the utility of the PanCancer Immune Profiling Panel generated with the Nanostring nCounter® Analysis System platform to provide information regarding immune profiles in the tumor microenvironment from tumor biopsy specimens that supports established immunohistochemical and cytometric results

3.0 Study Plan Overview

3.1. Description of Study Design, Population and Duration of Study Therapy

This is a phase I study of combination immunotherapy with IFN-γ and the PD-1 inhibitor nivolumab in patients with advanced solid tumors who have progressed on at least one prior systemic therapy, which may include prior immunotherapy. Patients will be treated with a one week induction phase (IP) of IFN-γ, followed by a combination phase (CP) with IFN-γ and nivolumab for three cycles, followed by a single agent phase of only nivolumab for up to one year. The study will primarily assess the safety and tolerability of the combination. Tumor assessments will occur after three cycles of combination therapy, then every three cycles thereafter. Secondary objectives including ORR, PFS, and OS will also be assessed, as will various correlative analyses. Initial accrual will occur in three cohorts of six patients each, in a dose escalation design to determine a recommended phase 2 dose (RP2D). Additional cohorts of 6 patients may be added to assess alternative dose levels based on safety and/or correlative data in the initial cohorts. Once endpoints for safety (using DLT criteria) are met, expansion cohorts will be opened for up to 15 patients.

4.0 Patient Selection Inclusion & Exclusion Criteria

4.1. Inclusion Criteria

Copyright© 2016 Fox Chase Cancer Center® Office of Clinical Research. All rights reserved.

4.1.1. All patients must have received at least one line of systemic therapy in the metastatic setting. Prior immunotherapy is allowed, including prior treatment with nivolumab,another PD-1 inhibitor, or a PD-L1 inhibitor, as long as the reason for discontinuation of a prior PD-1 pathway inhibitor was not for drug-related toxicity.

4.1.2. For dose escalation portion of the study: Patients must have a histologically or cytologically confirmed metastatic solid tumor that has shown clinical or pre-clinical evidence of responding to anti-PD-1 therapy or the capacity to up-regulate PD-L1. These tumor types may include but may not be limited to: RCC, UC, melanoma, NSCLC, small cell lung cancer, SCCHN, ovarian carcinoma, triple negative breast cancer, gastric cancer, microsatellite instability expressing (MSI-high) colon cancer, hepatocellular carcinoma, mesothelioma, gastrointestinal stromal tumors, endometrial carcinoma, liposarcomas, chondrosarcomas, and uterine sarcomas. Patients with solid tumor types not listed above may be enrolled at the discretion of the Principal Investigator.

4.1.3. For dose expansion cohorts: Dose expansion portion of study will include two separate cohorts.

a. One cohort will incorporate patients with esophageal, gastroesophageal junction (GEJ) or gastric carcinomas (squamous cell carcinoma or adenocarcinoma if predominant histology). These patients must have received at least one prior systemic therapy for metastatic disease. Patients who had prior neoadjuvant or adjuvant chemotherapy as part of curative intent primary therapy but recurred in less than 6 months would also be eligible. Patients with HER2+ disease must have received trastuzumab with disease progression prior to enrollment. Patients on this arm may have had prior treatment with drugs targeting the PD-1 pathway, but will be limited to a maximum of 5 patients.

b. The second cohort will include patients who have progressed on prior PD-1 pathway inhibition (single agent or in combination) in solid tumor types where these drugs are standard of care. The reason for discontinuation of the prior PD-1 pathway drug must not have been for toxicity. Eligible tumor types include melanoma, RCC, UC, NSCLC, and SCCHN, but subsequent tumor types wherein relevant agents become an approved standard of care would also become eligible..

4.1.4. Patients must have measurable disease per RECIST criteria v. 1.1 as described in detail in section 11.0.

4.1.5. Patients must have a site of disease that is amenable to pre-treatment and on-treatment core biopsies. At least 3 formalin fixed, paraffin embedded (FFPE) slides at five microns each may be collected at each biopsy. Determination of tissue accessibility and quantity will be made by the consenting clinician. Patients must consent to the two study-required biopsy procedures.

Copyright© 2016 Fox Chase Cancer Center® Office of Clinical Research. All rights reserved.

4.1.6. Age > 18 years.

4.1.7. Eastern Cooperative Oncology Group (ECOG) performance status 0 or 1.

4.1.8. Patients must have normal organ and marrow function as defined below:

Absolute neutrophil count (ANC)	> 1,500/mcL
Platelets	> 100,000/mcL
Total bilirubin	≤ 1.5 x upper normal limit (UNL)<br>(Except patients with Gilbert's syndrome, in whom total bilirubin must be <3.0 mg/dL)
AST/ALT (SGOT/SGPT)	< 3 times institutional normal limits
Creatinine	< 1.5 times the ULN<br>OR<br>Creatinine clearance > 40 mL/min (as measured or calculated by Cockroft-Gault formula)

4.1.9. Ability to understand and willingness to sign a written informed consent and HIPAA consent document.

4.2. Exclusion Criteria

4.2.1. Patients who have had anti-cancer systemic therapy within 2 weeks prior to entering the study. Radiation is allowed.

4.2.2. Patients may not have any active or recent history of a known or suspected autoimmune disease or recent history of a syndrome that required systemic corticosteroids or immunosuppressive medications, except for syndromes which would not be expected to recur in the absence of an external trigger. Subjects with vitiligo, type I diabetes mellitus, or residual hypothyroidism due to autoimmune thyroiditis only requiring hormone replacement are permitted to enroll.

4.2.3. Any condition requiring systemic treatment with corticosteroids (> 10 mg daily prednisone or equivalent) or other immunosuppressive medications within 14 days prior to first dose of study drug. Inhaled or topical steroids and adrenal replacement steroid doses > 10 mg daily prednisone or equivalent are permitted in the absence of active autoimmune disease.

4.2.4. Patients may not be receiving any other investigational agents.

4.2.5. Patients with known active or symptomatic central nervous system (CNS) metastases and/or carcinomatous meningitis. Asymptomatic, treated, and/or stable brain metastases, as measured by subsequent radiologic evaluations at least two months apart, are permitted.

4.2.6. History of allergic reactions attributed to compound of similar chemical or biologic composition to the agent(s) used in this study.

Copyright© 2016 Fox Chase Cancer Center® Office of Clinical Research. All rights reserved.

4.2.7. Uncontrolled intercurrent illness including, but not limited to, ongoing or active infection, symptomatic congestive heart failure, unstable angina pectoris, cardiac arrhythmia, uncontrolled hypertension or psychiatric illness/social situations that would limit compliance with study requirements.

4.2.8. Known human immunodeficiency virus (HIV) positive or history of acquired immune deficiency syndrome (AIDS) or AIDS-defining illness.

4.2.9. Known current or a history of hepatitis B or C virus, including chronic and dormant states, unless disease has been treated and confirmed cleared.

4.2.10. Any medical condition that in the investigator's opinion could interfere with interpretation of study or toxicity, or increase the risk to the patient related to potential toxicity.

4.2.11. Major surgery within 4 weeks of initiation of study drug.

4.2.12. Pregnant or breast feeding. Refer to section 4.4 for further detail.

4.2.13. A second invasive malignancy requiring active treatment.

4.3. Inclusion of Women and Minorities

Men and women, regardless of race, ethnic group or sexual orientation are eligible for this study

4.4. Pregnancy

The effects of nivolumab on the developing human fetus are unknown. IFN-γ is pregnancy category C, as it has shown an increase in the incidence of spontaneous abortions when given to primates at 100 times the dose given to humans. A study in pregnant primates treated with doses of IFN-γ at 2-100 times the normal human dose did not show any increase in teratogenic potential. However, for these reasons, women of child-bearing potential (WOCBP) and men must agree to use adequate contraception (hormonal or barrier method of birth control; abstinence) prior to study entry, for the duration of treatment, and for at least 3 months after the completion of treatment. Should a woman become pregnant or suspect she is pregnant while participating in this study, she must inform her treating physician immediately.

Prior to study enrollment, WOBCP must be advised of the importance of avoiding pregnancy during trial participation and the potential risk factors for an unintentional pregnancy. In additional, men enrolled on this study should understand the risks to any sexual partner of childbearing potential.

All WOCBP must have a negative pregnancy test within 72 hours prior to receiving the first dose of the investigational agent(s). If the pregnancy test is positive, the patient must not receive protocol treatment and must not be enrolled in the study.

WOCBP is defined as follows: Any female who has experienced menarche and who has not undergone successful surgical sterilization (hysterectomy, bilateral tubal

Copyright© 2016 Fox Chase Cancer Center® Office of Clinical Research. All rights reserved.

ligation, or a bilateral oophorectomy) or is not postmenopausal (defined as amenorrhea ≥ 12 consecutive months, or women on hormone replacement therapy (HRT) with documented plasma follicle-stimulating hormone (FSH) level > 35 mIU/ml). Even women who are using oral, implanted, or injectable contraceptive hormones or mechanical products (diaphragm, condoms, spermicides) to prevent pregnancy or practicing abstinence or where partner is sterile (e.g. vasectomy), should be considered to be WOCBP.

4.5. Patient Registration

Eligible participants will be entered on study centrally by the Fox Chase Cancer Center ERP Registrar or their designee. Following registration, participants must begin protocol treatment within 14 days of registration. Issues that would cause treatment delays must be discussed with the Sponsor Investigator. If a participant does not receive protocol therapy following registration, the participant will be recorded as withdrawn from study and will be replaced. The Study Monitor must be notified as soon as possible if a participant does not begin protocol treatment as scheduled.

Participants may be registered from 9:00 am to 5:00 pm excluding weekends and holidays by emailing FCCC.Monitor@fccc.edu. The site's investigator or designee will email the subject's initials and date of consent for registration. The study monitor will then issue a participant ID number.

After the screening phase, the completed registration form, consent and HIPAA signature pages and eligibility checklist will be emailed to the study monitor for enrollment. The study monitor will notify the site by email once enrollment is confirmed. Participants must be registered and have received a sequence number assigned by the study monitor prior to the initiation of treatment.

Exceptions to the current registration policies will not be permitted.

5.0 Treatment Plan

5.1. General Overview

Treatment will be administered on an outpatient basis as described below. Dose delays and modifications should follow protocol guidelines described in section 6.0. Consult https://www.opdivosafetytool.com/#/superhome for management of irAEs. Missed days will be made up as outlined in section 5.13. If treatment delays are > 42 days, study therapy will be discontinued.

5.2. Baseline Assessments

5.2.1. History and Physical

All patients will have a baseline history and physical (H&P) performed by a clinician prior to administration of the first dose of IFN-γ

5.2.2. Imaging

Copyright© 2016 Fox Chase Cancer Center® Office of Clinical Research. All rights reserved.

All patients will undergo baseline imaging with computed tomography (CT) of the chest, abdomen, and pelvis (CAP) ≤ 28 days from IP day. An optional nuclear medicine bone scan should be performed for patients with suspected or known bony metastatic disease ≤ 28 days from IP day 1.

All patients will have a baseline imaging study of the brain ≤28 days from IP day 1 with either:

a) An MRI with contrast, or
b) CT scan of the brain with and without contrast

5.2.3. Laboratory studies

Will include a complete blood count (CBC), a complete metabolic panel (CMP), and thyroid stimulating hormone (TSH) to be performed and reviewed ≤ 14 days prior to starting induction therapy with IFN-γ. There will also be a serum pregnancy test drawn for pre-menopausal females and all patients will undergo baseline screening for hepatitis B and C with hepatitis B surface antigen testing and either hepatitis C antibody or RNA testing.

5.3. Correlative Studies

5.3.1. Tumor Biopsy

All patients will undergo a baseline biopsy of a primary or metastatic site of disease within 21 days prior to initiation of treatment with IFN-γ on IP day 1.

5.3.2. Serum Correlatives

Peripheral blood for correlative studies will be drawn at the same time as baseline laboratory studies as per direction under Section 8.0 Correlative / Special Studies. These will include pharmacokinetic (PK) and antibody (Ab) studies of the investigational agents.

5.3.3. Other Correlatives

Peripheral fluid samples such as ascites or peritoneal fluid, pleural fluid, pericardial fluid or urine may be collected from certain patients for research purposes, including but not limited to flow cytometry, histocytopathology, and cytokine studies, if made available as part of standard clinical care for an individual patient.

5.4. Treatment Phases

5.4.1. Induction Phase

The first phase of treatment will begin with IFN-γ alone given every other day on IP days 1, 3, 5 and 7. These days will be denoted starting from IP day 1 as the first day of treatment through IP day 7 as the last drug administration day of the induction phase. Patients and/or caregivers will be trained on self-administration of subcutaneous IFN-γ on IP day 1, if necessary. Patients will

Copyright© 2016 Fox Chase Cancer Center® Office of Clinical Research. All rights reserved.

document each dose administered in a diary provided to them by the study coordinator.

Post-induction correlative blood tests will be drawn on C1D1, along with PK and antibody studies.

Patients in the dose escalation portion of the trial MUST have their second biopsy on C1D1 post-induction, but prior to the first dose of nivolumab. Ideally, the second biopsy will be performed at the same site as the initial baseline biopsy, but if this is deemed unsafe by the treating clinician an alternative site may be acceptable. If a good faith effort to obtain tissue via second biopsy is performed but unsuccessful for any reason, the patient may forgo this second biopsy and remain on study.

5.4.2. Combination Phase

The next phase of treatment will proceed with the addition of nivolumab along with continuation of IFN-γ. Patients will receive nivolumab, as illustrated below, every two weeks (14 days) during this phase, with a cycle lasting 28 days.

The first day will be denoted C1D1, on which day patients will perform supervised self-administration of IFN-γ in the morning at Fox Chase Cancer Center (FCCC) and will receive their first dose of nivolumab in the afternoon.

Post-induction serum correlatives will be secured on this day. PK studies for both IFN- γ and nivolumab will be drawn 4-8 hours after IFN-γ administration.

The first dose of nivolumab will be administered once the IFN-γ PK and pre-nivolumab PK have been drawn. The final nivolumab PK can be drawn after nivolumab completes. This will mean a long day for the patient. The only other day that this schedule will apply will be for C2D15. On all other days IFN- γ may be self-administered as usual by the patient and draws for correlative studies should not significantly impact the time the patient spends at the center.

The second dose of nivolumab will be given 14 days later, denoted C1D15, and the third dose will mark the start of cycle 2 (C2D1).

Patients in the dose expansion cohorts should get their second biopsy between the third and fourth doses of nivolumab, optimally on C2D8, but can be scheduled anytime from C2D8-C2D15.

Patients will be assessed for DLTs during this phase for 6 weeks, starting C1D1 until C2D14.

Tumor assessments with computed tomography of the chest, abdomen, and pelvis (CT CAP), as well as optional nuclear medicine bone scan per physician

Copyright© 2016 Fox Chase Cancer Center® Office of Clinical Research. All rights reserved.

discretion, will occur after three cycles (at the conclusion of the CP) on or about C4D1, then every three cycles thereafter. MRIs may be used in place of CT scans if necessary for renal function, contrast allergy, or deemed more appropriate by treating clinician.

Combination therapy will conclude after three cycles, or after C3D28, at which point IFN-γ will be stopped and patients who are clinically benefitting will continue to receive nivolumab as part of the single-agent phase.

5.4.3. Single Agent Phase

This phase will start with the dose of nivolumab given on C4D1.

Patients will continue to receive nivolumab if benefitting and will stop administering IFN-γ.

The schedule of nivolumab during this phase will change to every three weeks, so a cycle during this phase will be 21 days and include only 1 dose of nivolumab on D1 (Ex. C4D1, C5D1, etc.).

Patients may receive nivolumab if clinically benefitting for up to one year, equivalent to 15 doses during the single agent phase.

Patients who complete all doses of nivolumab through the combination and single agent phases will ultimately receive 21 doses (across 18 cycles) of nivolumab.

Tumor assessments will occur approximately every three cycles during this phase.

Patients may have the option of restarting IFN-γ during this phase if, in the opinion of the treating clinician and the PI, the patient had been experiencing clinical benefit during the combination phase but demonstrated evidence of clinical progression once proceeding to single agent nivolumab. Patients would restart at the same dose and schedule of IFN-γ last administered and continue until the end of the study.

5.5. Treatment Administration

Table 1: Overview of Investigational Agents

Agent	Dose	Route	Schedule	Phase Length
IFN-γ	Starting dose 50 mcg/m^2	SQ inj	Every other day	*Induction*: IFN-γ ONLY given IP day 1 to IP day 7

Copyright© 2016 Fox Chase Cancer Center® Office of Clinical Research. All rights reserved.

Nivolumab	*3 mg/kg	IV infusion over 60 minutes	*Combination*: Every 2 weeks	*Combination*: For 3 cycles starting C1D1 to C3D28
			Single Agent: Every 3 weeks	*Single Agent*: Nivolumab for up to 21 total doses (approximately 1 year / 18 cycles)

*Note: To remain consistent with FDA changes to labeling, Nivolumab dose will be 240 mg fixed dose for patients enrolling onto Amendment 2.

5.5.1. Prophylactic or Supportive Medications

IFN-γ
Acetaminophen 325 mg orally PRN per patient preference can be taken 30-60 minutes prior to injection for fevers and myalgias and may be used supportively at a dose of 325-650 mg orally Q6H PRN (≤ 3 gm/day).

Anti-depressant medication in the form of a selective serotonin re-uptake inhibitor (SSRI) or next generation anti-depressant medication may be taken orally by patient to start up to two weeks prior or at any point during therapy to treat or protect against the development of depression. This should be decided between the patient and treating clinician.

Ibuprofen 200 mg orally to 400 mg orally Q4-6H PRN (≤ 1200 mg/day) may be taken prophylactically or for supportive purposes for fevers and myalgias assuming adequate renal function as viewed by the treating clinician

Diphenhydramine 25 to 50 mg orally Q4-6H PRN (</= 300mg/day)

Nivolumab
None required

5.6. Dose Escalation Cohort

During the dose escalation portion of the study, patients will be enrolled to dose escalation cohorts at three dose levels of 6 patients each. Dose levels are defined in table 2 below.

Dose escalation determination will be based on assessment of dose-limiting toxicities (DLTs) occurring during the first six weeks of the combination phase.

Based on the number of DLTs seen in the cohort at the end of the six week toxicity window, the dose of IFN-γ in subsequent cohorts will be either escalated or de-escalated as defined per definition of DLTs in section 6.4 below. The dose of nivolumab will remain fixed throughout the study.

After a cohort of six patients has begun treatment, the study will be on hold to further accrual while that group of patients is monitored for DLTs.

Copyright© 2016 Fox Chase Cancer Center® Office of Clinical Research. All rights reserved.

Once all six patients at a dose level have completed the six week DLT assessment phase, the next dose level can begin accrual, either higher or lower based on the rules provided in table 3. If any dose level results in two patients with DLTs prior to completing accrual, it would meet criteria to de-escalate to the next dose level.

The dose escalation phase will plan to evaluate at least three dose levels of IFN-γ in combination with nivolumab. The highest dose level cohort tested that has ≤ 1 DLT out of six patients will be denoted the maximum tolerated dose (MTD). Only those patients that have completed the week of IFN-γ induction and have received at least one dose of nivolumab will be considered evaluable for the primary DLT assessment. Patients that stop the trial for any reason other than a DLT during the six week toxicity window may be replaced for analysis of the primary endpoint.

While a MTD will be defined, the PI and members of the dose escalation committee defined above reserve the right to open dose expansion cohorts of 6 patients at a dose of IFN-γ lower than the defined MTD based on cumulative toxicity information outside the DLT window or correlative evidence suggesting a greater immunogenic effect of IFN-γ at lower doses. The lower dose may be defined as the RP2D based upon committee assessment of the safety, efficacy, and correlative data.

Once the RP2D dose has been established, the disease-specific expansion cohorts may begin accrual. Decisions on de-escalation and dose expansion will be made by a committee consisting of the PI, sub-investigators, study coordinators, and statistics team, with input from the institutional disease safety monitoring committee. Any decision will be documented in meeting minutes.

Detailed instructions for dose escalation and rules for opening of expansion cohorts are provided in table 3, while the operating characteristics of the 6 patient cohort design appear in table 4.

Copyright© 2016 Fox Chase Cancer Center® Office of Clinical Research. All rights reserved.

Table 2: Dose Levels of IFN-γ

Dose Level	Dose (every other day)
-2	15 mcg/m^2
-1	25 mcg/m^2
1 (Starting Dose)	50 mcg/m^2
2	75 mcg/m^2
3	100 mcg/m^2

Table 3: Decision Rules for Dose Escalation and Cohort Expansion

Number of Patients with DLT	Escalation and Expansion Decision Rules
0-1 out of 6	• If first or second dose level evaluated, escalate to next highest dose level<br>• If third dose level tested (or more), declare this dose the RP2D and proceed to opening expansion cohorts
$\geq$2 out of 6	• Consider this dose unacceptable. De-escalate dose level and open new expansion cohort of six patients at next lowest dose level<br>• If third cohort (dose level -2), terminate study

Table 4: Operating Characteristics 6+6 design

True DLT rate	Chance dose is considered safe	Chance considered too toxic
0.05	0.97	0.03
0.10	0.89	0.11
0.15	0.78	0.22
0.20	0.66	0.34
0.25	0.53	0.47
0.30	0.42	0.58
0.35	0.32	0.68
0.40	0.23	0.77
0.45	0.16	0.84
0.50	0.11	0.89
0.55	0.07	0.93

5.7. Expansion Cohorts

The RP2D will be expanded into two separate disease-specific cohorts of 15 patients each. One cohort will incorporate patients with esophagogastric carcinomas (squamous cell carcinoma or adenocarcinoma) who have received at least one prior systemic therapy for metastatic disease. Patients who recurred in less than 6 months after prior curative intent systemic therapy would also be eligible. The second expansion cohort will include patients who have progressed on prior PD-1 pathway inhibition (single agent or in combination) in solid tumor types where these drugs are standard of care.

Copyright© 2016 Fox Chase Cancer Center® Office of Clinical Research. All rights reserved.

Eligible tumor types may include melanoma, RCC, UC, NSCLC, and SCCHN, but subsequent tumor types wherein relevant agents become an approved standard of care would also become eligible. This expansion is designed to further assess safety and preliminary efficacy in two treatment cohorts where this combination would be most likely to be clinically relevant.

Early stopping for rules for excess toxicity in the expansion cohorts will be applied in order to account for that possibility in each disease-specific expansion cohort. A toxicity event for the purposes of safety stopping is defined as per the definitions of a DLT denoted in table 5 due to the combination of IFN-γ and nivolumab. We assume that a 20% rate of toxicity events would be acceptable and a 40% rate of toxicity would be unacceptable. Using a likelihood ratio approach, we define early stopping based on significant evidence in favor of a 40% toxicity rate vs. 20% toxicity rate with a likelihood ratio of 4. Based on these criteria, the stopping rules are defined below in table 5.

Table 5: Early stopping rules for disease specific expansion cohorts

Stop if:	Proportion of patients with toxicities	Likelihood ratio
3 toxicities in 5 or fewer patients	0.60	≥4.5
4 toxicities in 8 or fewer patients	0.50	≥5.1
5 toxicities in 12 or fewer patients	0.42	≥4.3
6 toxicities in 15 or fewer patients	0.40	≥4.8

While on study, patients may undergo up to two intra-patient dose reductions of IFN-γ for toxicities meeting criteria in section 6.0 below. Patients may stop IFN-γ completely and continue on nivolumab after two dose reductions if otherwise tolerable and/or clinically benefitting as deemed by treating clinician. If patients recover to grade < 2 while off IFN therapy, but are otherwise deemed to be clinically benefitting, they may continue to receive single agent nivolumab for the duration of the trial, or as per other stopping rules as outlined in section 5.13.

5.8. Recommended Phase 2 Dose Definition

The final dose level during the dose escalation phase that yields ≤ 1 DLTs will define the RP2D selection for further study

5.9. Concomitant Medications, Supportive Care, Excluded Therapies and Restrictions

5.9.1. Supportive Care

Palliative radiation therapy is permitted for irradiating metastatic sites causing pain, bleeding, or deemed by the treating clinician to pose a clinically meaningful risk to the patient at that time or in the future that cannot be managed adequately using systemic or local therapies, as long as the treating clinician feels the patient is otherwise clinically benefitting and would more likely than not continue to garner benefit at the conclusion of radiation. If radiation is felt to be necessary during the combination or single agent phase,

Copyright© 2016 Fox Chase Cancer Center® Office of Clinical Research. All rights reserved.

the study agents may be held during radiation therapy at the discretion of the treating clinician. If held, the drug(s) must be resumed within 42 days. However, patients are allowed to continue to receive study therapy during radiation treatment.

Surgical metastasectomy, or surgical resection of a solitary site of metastatic disease, is permitted for proven or presumed metastatic lesions that are causing intractable symptoms and/or growing discordantly from other sites of disease. Elective metastasectomy should be performed after completion of the combination phase if the treating clinician feels the patient is otherwise clinically benefitting and would more likely than not continue to derive benefit following successful surgical resection. Nivolumab should be held during the perioperative setting as clinically indicated and must be resumed within 42 days of last dose.

Patients may receive supportive blood products or antibiotics when appropriate.

Patients requiring other supportive or adjunctive therapies, including but not limited to percutaneous drainage or biliary or genitourinary stent placement, may be considered on an individual patient basis to be permitted to hold investigational therapy for up to 42 days.

Because of the potential for clinically relevant irAEs and the importance of prompt and aggressive corticosteroid therapy, a standardized algorithm should be followed. Please refer to https://www.opdivosafetytool.com/#/superhome for any suspicion of an irAE.

Patients unable to resume dosing after a 42 day hold will be removed from study and not replaced, unless they are in a dose escalation cohort and meet the criteria established in Section 5.6 regarding replacement of patients for assessment of DLT.

5.9.2. Concomitant Medications and Excluded Therapies

IFN-γ:
No known concomitant therapies warrant special attention

Nivolumab
Systemic corticosteroids > 10 mg prednisone (or equivalent), unless being used to treat a drug-related AE or adrenal insufficiency present prior to study enrolment, are prohibited

Any immunosuppressive agent that when given concomitantly with nivolumab may adversely affect the drug's efficacy is prohibited

Copyright© 2016 Fox Chase Cancer Center® Office of Clinical Research. All rights reserved.

Use of anti-neoplastic or anti-tumor agents not part of the study therapy, including chemotherapy or other immunotherapy, is not permitted while participating in the study.

Hormonal anticancer therapy is allowed

Use of concurrent investigational agents is not permitted

Copyright© 2016 Fox Chase Cancer Center® Office of Clinical Research. All rights reserved.

5.10. Duration of Therapy

Induction Phase (IFN-γ ONLY): 1 week, denoted IP day 1 to IP day 8

Combination Phase: maximum 3 cycles

Single agent phase (Nivolumab ONLY): up to completion of 1 year of total therapy or equivalent to 18 cycles or 21 total doses

5.11. Duration of Follow up

All patients will be followed for response and survival approximately every 3 months (+/- 14 days) for up to 2 years from study initiation or until death, whichever comes first.

Any patients who experience adverse events should be followed until one of the following occurs:

- Resolution of the adverse event to ≤ Grade 1 or baseline severity
- Determination that the adverse event is stable and is unlikely to resolve
- Initiation of another anti-cancer treatment

5.12. Treatment Beyond Disease Progression

Data from previous trials has clearly demonstrated a phenomenon in which patients treated with PD-1 inhibitors may derive clinical benefit despite initial radiologic evidence of disease progression.[63]

Accordingly, patients will be permitted to continue treatment beyond initial investigator-assessed progression as long as they meet the following criteria:

- Investigator-assessed clinical benefit
- Patient is tolerating treatment

All decisions to treat beyond progression must be approved by the PI and the rationale must be clearly documented within the study records.

Upon radiographic or clinical evidence of second progression, defined as an additional 20% or greater increase in total sum of tumor burden, patients must discontinue study therapy

Copyright© 2016 Fox Chase Cancer Center® Office of Clinical Research. All rights reserved.

5.13. Criteria for Discontinuation

5.13.1. General Reasons for Discontinuation or Withdraw from Study

In the absence of treatment delays due to adverse events, treatment may continue as above or until one of the following criteria applies:

- Disease progression
- Intercurrent illness that prevents further administration of treatment
- Unacceptable adverse events
- Treatment held > 42 days, unless meets criteria in section 5.13.3
- Patient becomes pregnant
- Patient decides to withdraw from the study
- General or specific changes in the patient's condition that render the patient unacceptable for further treatment in the judgment of the investigator

5.13.2. Discontinuation Criteria from IFN-γ (IP only)

- Any grade 2 drug-related cardiac condition or manifestation including ischemia, congestive heart failure or newly diagnosed arrhythmia
- Any grade 3 drug-related neurologic sequelae including altered mental status, gait disturbance, or seizure, if not explainable by another treatable and correctable cause

5.13.3. Discontinuation Criteria from Nivolumab (CP or single-agent phase)

- Any grade 3 drug-related uveitis or eye pain or blurred vision that does not respond to topical therapy and does not improve to grade 1 severity within the re-treatment period OR requires systemic treatment
- Any grade 3 non-skin, drug-related AE not improving to < 2 for > 5 days following initiation of appropriate supportive and corticosteroid and/or other appropriate medical therapy, with the following specific exceptions/guidelines for pneumonitis, adrenal insufficiency, neurologic toxicity, hypersensitivity reactions, liver function test abnormalities, and infusion reactions
- Grade 3 drug-related pneumonitis, adrenal insufficiency, neurologic toxicity or sequelae (including seizure), hypersensitivity reaction, or infusion reaction of any duration requires discontinuation
- Grade 3 drug-related laboratory abnormalities do not require treatment discontinuation except for following:
    - Grade 3 drug-related thrombocytopenia > 7 days or associated with clinically significant bleeding
    - Drug-related liver function test (LFT) abnormality meeting the following criteria:

Copyright© 2016 Fox Chase Cancer Center® Office of Clinical Research. All rights reserved.

  - AST or ALT > 5x the upper limit of normal (ULN)
  - Total bilirubin > 3x the ULN
- Any grade 4 drug-related toxicity or laboratory abnormality
- Any recurrence of a grade 3 toxicity of any type requires discontinuation
- Inability to reduce corticosteroid dose to 10 mg or less of prednisone or equivalent per day within 42 days
- Any dosing interruption lasting > 42 days, unless the PI is consulted and agrees with rationale and extenuating circumstances for resuming therapy after this time period. Tumor assessments must continue per protocol if dosing is interrupted.
- Nivolumab may be postponed for up to 5 business days due to weather emergencies, unplanned missed appointments, holiday or other unplanned events not related to toxicity. Nivolumab treatment will resume on the next available business day and continue on schedule in the event of a postponed treatment as described above.
- Any adverse event, laboratory abnormality, or intercurrent illness which, in the judgment of the PI or treating clinician, presents a substantial clinical risk to the subject with continued dosing.

The reason for study removal and the date the patient was removed must be documented in the medical record.

6.0 Dose Modifications

6.1. General Principles

Patients treated initially were dosed at the original FDA-approved dose of 3 mg/kg. Any patient who began at this dose remained at this dose. Upon approval of Amendment 2 of the protocol, subsequently accrued patients will be treated at the new FDA-approved fixed dose of 240 mg.

During the combination phase, missed doses of nivolumab may be postponed for up to 5 business days on up to 3 occasions in an individual patient due to weather emergencies, unplanned missed appointments, holiday or other unplanned events not related to toxicity

During the single agent phase, nivolumab administration may be altered by up to one calendar week in an individual patient due to weather emergencies, unplanned missed appointments, holidays or other unplanned events not related to toxicity

Dose reductions for IFN-γ are permanent, there are no dose re-escalations. Patients requiring > 2 dose reductions of IFN-γ will discontinue IFN-γ and may continue treatment on nivolumab if clinically benefitting. Missed doses of IFN-γ are not to be made up and patients will remain on every other day schedule as originally outlined.

Copyright© 2016 Fox Chase Cancer Center® Office of Clinical Research. All rights reserved.

Patients requiring treatment to be held > 42 days for recovery from toxicity or adjunct palliative therapy (radiation, surgery, etc. as described in section 5.9.1 above) must discontinue protocol treatment unless meet criteria defined above.

6.2. Dose Level Adjustment –

Patients in the dose escalation cohort will receive IFN-γ induction and combination treatment at the IFN-γ doses described in Table 2 above.

Patients in the dose escalation cohort experiencing a DLT at a given IFN-γ dose may de-escalate to the next lower IFN-γ dose and continue on treatment as long as not meeting criteria for discontinuation.

6.3. Toxicity Assessment and Grading

Comprehensive assessments of drug-related adverse events (AEs) experienced by the patient will be performed throughout the course of the study.

Anticipated and previously reported adverse drug reactions are described below and at https://www.opdivosafetytool.com/#/superhome.

Toxicity and event severity will be graded using the National Cancer Institute (NCI) Common Toxicity Criteria for Adverse Events (CTCAE) version 4.03 grading system and appropriate clinical management will be provided based on sound clinical judgment by the treating clinician and with reference to the nivolumab AE guidelines available at https://www.opdivosafetytool.com/#/superhome.

6.4. Definition of DLTs

DLTs will be assessed during the first 6 weeks of the combination phase of the study (from C1D1 to C2D14) for patients in the dose escalation cohorts.

Any of the following AEs occurring during the DLT observation period of the combination phase attributable to one or both study drugs will be classified as a DLT, as defined in table 6. Dose-limiting toxicities include all treatment-emergent AEs of the specified grades, regardless of investigator attribution or relatedness. Toxicities with a clear alternative explanation (eg, due to disease progression or unrelated non-medical event) can be deemed a non-DLT.

Subjects whose treatment is discontinued because of a DLT must be followed until resolution or stabilization of the DLT event, whichever comes first.

Table 6: Definition of DLTs (per NCI CTCAE v4.03)

Hematologic	Grade 4 neutropenia
	Febrile neutropenia, defined as ANC $\leq$ 500/mm^3 and fever >100.4°F (38.0°C)
	Grade $\geq$ 3 thrombocytopenia with clinically significant bleeding
	Grade 4 thrombocytopenia lasting > 7 days

Copyright© 2016 Fox Chase Cancer Center® Office of Clinical Research. All rights reserved.

Non-Hematologic	Any grade 4 toxicity
	Grade ≧ 3 nausea, vomiting, or diarrhea that does not improve to grade < 2 within 72 hours despite maximal medical therapy
	Grade ≥ 3 rash that does not improve to grade < 2 within 7 days despite maximal medical therapy, unless involves desquamation, mucosal involvement, or systemic steroids
	Grade ≧ 3 drug-related uveitis, iritis, episcleritis, eye pain, or blurred vision
	Any other grade ≧ 3 non-laboratory or symptom-based toxicity
Laboratory Values	Grade ≥ 3 non-hematologic laboratory value that does not return to grade 1 despite maximal medical therapy OR that requires hospitalization
Other	Inability to complete the first 3 doses of nivolumab

6.5. Instructions for Specific Dose Modifications

In the event of significant toxicity, doses may be delayed and/or reduced (IFN-γ only) as described below in table 7.

In the case of multiple toxicities, dose modification will be based on the worst toxicity observed.

Patients who have nivolumab held for toxicity should also hold IFN-γ during that same time period. Once decision made to restart nivolumab, IFN-γ may be restarted at same dose and schedule, making sure IFN-γ dosing occurs on days of nivolumab administration.

Dose modifications for AEs documented during the combination phase will be selected based on the option in table 7 below that is more conservative.

Table 7: Dose Modifications

**Toxicity**	**NCI CTCAE Grade**	**IFN-γ**	**Nivolumab**
**Hematologic Laboratory Abnormalities**	Grade 1-3	No change, unless thrombocytopenia with clinically significant bleeding, then same as grade 4	No change
	Grade 4	Discontinue therapy	Discontinue therapy
**Non-Hematologic**<br><br>**Non-Laboratory**	Grade 1	No change	No change
	Grade 2 cardiac ischemia, new arrhythmia, congestive heart failure	Discontinue medication	No change
	Grade 2 diarrhea, colitis,	. No change if being	Hold drug until improves to grade ≤

Copyright© 2016 Fox Chase Cancer Center® Office of Clinical Research. All rights reserved.

	pneumonitis, hypophysitis, nephritis, creatinine elevation, or adrenal insufficiency	given alone. Hold IFN-γ while nivolumab held.	1. Administer maximal supportive therapies and consider systemic corticosteroid therapy. If corticosteroids added, taper over 28 days before restarting drug
	Other grade 2	No change	Consider holding dose and instituting corticosteroid therapy for persistent symptoms until resolves to grade < 2
	Grade 3 diarrhea, colitis, hypophysitis, nephritis, creatinine elevation, or rash	If controllable with supportive medications and reduce to grade ≤ 2, continue at same dose. Otherwise, hold until recovery to grade ≤ 2 and must reduce one dose level. Hold IFN-γ while nivolumab held.	Hold drug, initiate supportive therapy and consider immediately adding corticosteroid therapy until resolves to grade < 2 or baseline. If corticosteroids added, taper over 28 days before restarting drug. If no improvement despite > 5 days of systemic corticosteroid therapy, discontinue drug permanently and consider adding non-corticosteroid immunosuppressive
	Grade 3 pneumonitis or adrenal insufficiency	Discontinue medication	Discontinue medication
	Grade 3 drug-related neurologic sequelae including seizure, altered mental status, or gait disturbance	Discontinue medication	Discontinue medication
	Grade 3 infusion or hypersensitivity reaction	Discontinue medication	Discontinue medication
	Other Grade 3	If controllable with supportive medications and reduce to grade ≤ 2, continue at same dose. Otherwise, hold until recovery to grade ≤ 2 and must reduce one dose level. Hold IFN-γ while nivolumab held.	Hold drug, initiate supportive therapy and consider immediately adding corticosteroid therapy until resolves to grade < 2 or baseline. . If corticosteroids added, taper over 28 days before restarting drug. If no improvement despite > 5 days of systemic corticosteroid therapy, discontinue drug permanently and consider adding non-corticosteroid immunosuppressive
	ANY Grade 3		Second recurrence of ANY grade 3 toxicity, discontinue medication
	Grade 4	Discontinue therapy	Discontinue therapy and immediately institute corticosteroid

Copyright© 2016 Fox Chase Cancer Center® Office of Clinical Research. All rights reserved.

			or immunosuppressive therapy as per guidelines for management of irAE present available in table in appendix 1

Copyright© 2016 Fox Chase Cancer Center® Office of Clinical Research. All rights reserved.

**Laboratory Abnormalities**	Grade 1	No change	No change
	Grade 2 Liver Function Test Abnormalities (ALT or AST 3-5x ULN TBili 1.5- 3x ULN)	No change if being given alone. Hold IFN-γ while nivolumab held.	Hold drug and administer systemic corticosteroids per guidelines until resolves to grade 0 or 1. Taper steroids over 28 days before restarting drug
	Grade 3 Thrombocytopenia	Hold IFN-γ until resolves to grade < 2 and reduce one dose level if given alone. Hold IFN-γ while nivolumab held.	Hold drug until resolves to grade < 2. If no improvement > 7 days despite medical management, discontinue
	Grade 3 Liver Function Test Abnormalities (ALT or AST > 5x ULN TBili > 3x ULN)	Hold IFN-γ until resolves to grade < 2 and reduce one dose level if given alone. Hold IFN-γ while nivolumab held.	Discontinue therapy and consider immediately instituting corticosteroid or immunosuppressive therapy as per guidelines. Patients may restart treatment if elevation demonstrated due to another cause and returns to grade ≤ 1
	Grade 3 (not otherwise specified)	Treat with appropriate medical therapy and hold drug until resolves to grade ≤ 2. If controllable with supportive therapy, may continue same dose. Hold IFN-γ while nivolumab held.	Hold dose and medically treat as appropriate. Restart when grade ≤ 2
	Grade 4	Discontinue therapy	Discontinue therapy

6.5.1. Treatment of Drug-Related Infusion Reactions

IFN-γ- not applicable

Nivolumab: this agent contains only human immunoglobulin protein sequences and thus rarely leads to infusion hypersensitivity reactions. However, if one were to occur, it would manifest with fevers, chills, rigors, pruritus, alterations in blood pressure, bronchospasm, or other symptoms. Treatment recommendations should follow guidelines per table 8

Copyright© 2016 Fox Chase Cancer Center® Office of Clinical Research. All rights reserved.

Table 8: Assessment and Management of Nivolumab Infusion Reactions

NCI CTCAE Grade	Treatment	Subsequent Infusion Pre-medications
Grade 1	Infusion interruption not indicated. Monitor patient until recovery from symptoms	Diphenhydramine 50 mg<br>Acetaminophen 325 mg<br><br>Administer 30 minutes prior to nivolumab infusion
Grade 2	Stop infusion. Administer IV normal saline, diphenhydramine 50 mg IV and acetaminophen 325-1000 mg and monitor patient until resolution of symptoms. Provide corticosteroid or bronchodilator therapy as appropriate.<br>May restart infusion at 50% rate when symptoms resolve. If no further complications, may increase rate back to 100%. If symptoms do recur, treat as above and no further nivolumab will be administered at that visit.	Diphenhydramine 50 mg<br>Acetaminophen 325 mg<br>Corticosteroids (up to 25 mg IV hydrocortisone or equivalent)<br><br>Administer 30 minutes prior to nivolumab infusion
Grade 3-4	Immediately discontinue infusion. Begin IV infusion of normal saline along with diphenhydramine 50 mg IV, methylprednisolone 100 mg IV (or equivalent). Start bronchodilators and epinephrine 0.2 to 1 mg of a 1:1000 solution (for SQ administration) or 0.1 to 0.25 mg of a 1:10,000 solution (for IV administration) as needed. Monitor patient for recovery and until clinician is comfortable symptoms will not recur, including overnight hospital stay if warranted. Permanently discontinue medication.	None. Discontinue further treatments

7.0 Study Drugs

7.1. IFN-γ Formulation, Product identification, Package and Labeling

7.1.1. Product description

IFN-γ is a cytokine or biologic response modifier that exists as a single-chain polypeptide containing 140 amino acids. Production of this agent is achieved via fermentation of a genetically engineered *Escherichia coli* bacterium containing the DNA which encodes for the human protein. The agent is a sterile, clear, colorless solution pre-filled in a single-use vial for subcutaneous injection

Copyright© 2016 Fox Chase Cancer Center® Office of Clinical Research. All rights reserved.

Each 0.5 mL vial contains 100 mcg if IFN-γ formulated in 20 mg of mannitol, 0.37 mg disodium succinate hexahydrate, 0.14 mg succinic acid, 0.05 mg polysorbate 20 and sterile water

7.1.2. Availability

IFN-γ for this protocol will be supplied Horizon Pharma, LLC.

7.1.3. Solution preparation

Available as described above and per product label. Distributed as single use vials and designated amount per dose to be administered by patient

7.1.4. Storage requirements and Stability

Vials should be refrigerated at 2-8°C (36-46°F). If exposed to room temperature for greater than 12 hours, vials should be discarded. Do not freeze. Avoid shaking or excessive vigorous activity

7.1.5. Route of administration

Will be self-administered by patients or representative SQ

Note: Patients will be trained on appropriate administration technique and appropriate documentation of self-administration in the patient diary

7.2. Nivolumab

7.2.1. Product Description

Nivolumab is a human IgG4 PD-1 inhibitor

7.2.2. Availability

Will be purchased commercially for study use

7.2.3. Solution preparation

As per product label, required volume of drug to be withdrawn and diluted with 0.9% normal saline or 5% dextrose solution to final desired concentration

7.2.4. Storage Requirements and Stability

Store under refrigeration at 2-8°C (36-46°F) and protect from light by storing in original package until time of use. Do not freeze or shake.

7.2.5. Route of Administration

Will be administered as an IV infusion over 60 minutes

7.3. Drug Ordering, Storage and Handling

Copyright© 2016 Fox Chase Cancer Center® Office of Clinical Research. All rights reserved.

Following submission and approval of the required regulatory documents, participation in the study initiation meeting and receipt of the site activation letter from the CTO Protocol Development Project Manager, the initial order may be placed. Drug order forms and ordering procedure will be presented at the site initiation meeting.

7.4. Destruction of Drug

During the course of the study, any used vials of study drug will be destroyed at the site per Institutional SOPs after appropriate documentation of accountability. At the time of study closure, any unused and expired study drug will be destroyed at the site per Institutional SOPs after appropriate documentation of accountability unless otherwise specified.

7.5. Records to be kept at Site; Dispensing and Accountability

It is the responsibility of the Investigator to ensure that a current record of investigational product disposition is maintained at each study site where investigational product is inventoried and disposed. Records must comply with applicable regulations and guidelines, and should include:

- Amount received and placed in storage area.
- Amount currently in storage area.
- Label ID number or batch number.
- Dates and initials of person responsible for each investigational product inventory entry/movement.
- Amount dispensed to and returned by each patient, including unique patient identifiers.
- Amount transferred to another area for dispensing or storage.
- Non-study disposition (e.g., lost, wasted, broken).

8.0 Correlative /Special Studies

8.1. Biopsy Specimen Analysis

Purpose
Evaluation and assessment of effect on tumor and tumor microenvironment as compared to fresh baseline sample

Timing and number of specimens
Two samples will be collected per patient. All patients will undergo a baseline biopsy within 21 days prior to initiation of the induction phase of IFN-γ

Patients in the initial dose escalation portion of the study will undergo a second biopsy at the conclusion of the induction phase but prior to starting treatment with nivolumab (C1D1)

Patients in the dose expansion potion of the study will undergo their second biopsy after the third dose of nivolumab but prior to the fourth dose (C2D8-C2D14)

Copyright© 2016 Fox Chase Cancer Center® Office of Clinical Research. All rights reserved.

Please see study calendar in section 9.0 for further detail

Sample Procurement
Collection will be via core needle biopsy of primary or metastatic site. Same site should be biopsied on two occasions when possible. At least three core biopsy samples should be collected if possible for each individual procedure

Handling and Preparation
Specimens will be collected and sent immediately to the pathology department at FCCC to be prepared for evaluation as three FFPE 5 micron slides

Method of assessment:
Samples will be evaluated by at least three methods possibly including but not limited to: Simultaneous Multi-Channel Immunofluorescence (SMI), immunohistochemistry for markers such as PD-L1, MHC Class I, pSTAT1, CD45, and CD8, and Nanostring nCounter®

Detailed instructions for tissue collection, storage, processing, and shipment are provided in the laboratory manual

Statistical Analysis
Descriptive statistical analysis comparing initial and subsequent biopsy specimens will be guided by the FCCC Biostatistics Facility.

Comparisons between pre and post-treatment samples will be expressed by graphic display and a paired t-test may be used to determine if there is a statistically significant change.

8.2. Peripheral Blood Analysis

Purpose
Evaluation of samples to assess the effects of IFN-γ with and without nivolumab on various cytokines, receptors and cellular markers in order to illustrate proof-of-concept, investigate potential biomarkers of response, and understand changes in the host as a function of this combination therapy.

Timing and number of specimens
Three heparinized tubes of peripheral blood will be drawn from each patient at four time points. Please see study calendar in section 9.0 and laboratory manual for further detail.

Detailed instructions for peripheral blood collection, handling tissue collection, storage, processing, and shipment are provided in the laboratory manual.

Methods of assessment

Copyright© 2016 Fox Chase Cancer Center® Office of Clinical Research. All rights reserved.

Whole blood and plasma will be analyzed by several laboratory procedures available at FCCC in the laboratories of Dr. Siddharth Balachandran and Dr. Kerry Campbell. Complete detail of the study procedures can be accessed in the laboratory manual.

Statistical Analysis
Flow cytometry data will be compared and summarized using descriptive statistics, guided by FCCC Biostatistics Facility.

Staining levels expressed as scores will be tabulated categorically. Changes over time between samples will be portrayed graphically and further analyses including but not limited to paired t-tests may be used to evaluate for statistically significant changes.

8.3. PK and Neutralizing Antibody Assessments

Purpose
To gain an understanding of the PK effect of IFN-γ in this patient population

To understand the PK profile of nivolumab when given in combination with IFN-γ in comparison to published effects as a single agent

To evaluate whether either therapeutic agent stimulates a detectable host antibody response

Timing and number of specimens
See study calendar in section 9.0 for detail. Detailed instructions for these analyses including collection, handling, storage, processing, and shipment are provided in the laboratory manual. These will be supervised by Horizon Pharma and performed by a third party laboratory.

8.4. Other Specimens (Pleural Fluid, Peritoneal Fluid, Pericardial Fluid, Urine)

Purpose
To potentially study how treatment may affect changes or development of various bodily fluids, particularly in patients who develop new ascites or pleural/pericardial effusions while on treatment.

Specimens
Specimens collected as normal standard of care may be used for research purposes and will be collected and analyzed as outlined in the lab manual and managed by the PSL

Copyright© 2016 Fox Chase Cancer Center® Office of Clinical Research. All rights reserved.

9.0 Study Calendar

	Pre-Study [b]	IP D1	C1 D1	C1 D8	C1 D15	C1 D21	C2 D1	C2 D8	C2 D15	C3 D1	C3 D15	C4 D1[i]	C5-18 D1	Discontinuation [a]	Follow-up [m]
Informed consent & HIPAA	X														
Height	X														
β-HCG, HBV sAg, HCV Ab or RNA	X [d]														
Medical history	X	X			X		X		X	X	X	X	X		X
Physical exam	X				X		X		X	X	X	X	X		X
Concurrent meds	X	X			X		X		X	X	X	X	X		X
Vital signs (T, P, R, BP)	X	X			X		X		X	X	X	X	X		X
Weight	X	X			X		X		X	X	X	X	X		
Performance status	X	X			X		X		X	X	X	X	X		
CBC w/diff, plts	X			X	X	X	X		X	X	X	X	X		
CMP [c]	X			X	X	X	X		X	X	X	X	X		
Tumor Biopsy	X [e]							X [h]							
AE evaluation		X			X		X		X	X	X	X	X	X	
TSH	X									X			X [j]		
Radiologic evaluation [p].	X											X	X [k]		X [k]
Peripheral Blood Correlative Studies [o]	X		X						X				X [l]		
PK Correlates: IFN-γ [o]	X		X				X		X			X			
PK Correlates: Nivolumab [o]	X		X				X		X			X	X [l]		X
Antibody Correlates: IFN-γ [o]	X						X					X	X [l]		
Antibody Correlates: Nivolumab [o]	X						X					X	X [l]		X [n]
IFN-γ Dose		X [f]	X		X		X		X	X	X				
Nivolumab Dose			X		X		X		X	X	X	X	X		

Copyright© 2016 Fox Chase Cancer Center® Office of Clinical Research. All rights reserved.

a: To occur 30-42 days after discontinuation of study treatment

b: Pre-study H&P and all labs must be < 14 days prior to registration. Tumor measurements and radiologic evaluations must be < 28 days prior to IP day 1.

c: Albumin, alkaline phosphatase, total bilirubin, bicarbonate, BUN, calcium, chloride, creatinine, glucose, LDH, phosphorus, potassium, total protein, SGOT [AST], SGPT [ALT], sodium.

d: Serum pregnancy test (women of childbearing potential) must be completed < 72 hours before registration.

e: Baseline, pre-treatment biopsy must be performed < 21 days of starting treatment on IP day 1.

f: IFN-γ will be administered by patient or representative under supervision for first dose on IP day 1. Subsequent dosing will be self-administered by patient at home on every other day basis through end of combination phase unless schedule change coordinated by clinician and study coordinator, except on C1D1 when self-administered at FCCC to facilitate timing of PK and antibody procurement. The clinician or designee will train patient or representative on appropriate administration technique and appropriate documentation of self-administration in the diary.

g: Biopsy (if possible from same site previously sampled) will be performed on C1D1 for all patients enrolled to dose escalation cohort. H&P, med evaluation, vital signs, PS assessment, CBC, CMP, and AE evaluation may also be performed on C1D1.

h: Biopsy (if possible from same site previously sampled) will be performed between C2D8-C2D14 for all patients in the expansion cohorts.

i: C4D1 marks the beginning of the single agent phase, at which point nivolumab will be given every 3 weeks. Patients will thus be seen on D1 of each cycle starting at C4.

j: TSH will be drawn every odd numbered cycle starting at C5 during the single agent phase.

k: Radiologic assessments will be performed every 3 cycles during the single agent phase on D1 of the cycle (C7, C10, C13, C16), and then three weeks after the final dose of nivolumab, on what would have otherwise been C19D1. Images will be obtained for each cycle within a five day window.

l: Antibody studies for IFN-γ and nivolumab and PK for nivolumab , as well as samples for final comparative peripheral blood correlatives, will be drawn once during the single agent phase on C7D1 or C8D1.

m: Follow-up for disease progression. Evaluation for resolution of treatment related toxicities and assessments for survival should be conducted every 3 months for 2 years.

n: One blood draw for antibodies to nivolumab will be drawn during the follow-up period 2-4 months after the final dose of nivolumab

o: Please see lab manual for precise timing and schedule for correlative, PK, and antibody collections

p: CT CAP and brain CT / MRI for all patients. Bone scan if indicated

Copyright© 2016 Fox Chase Cancer Center® Office of Clinical Research. All rights reserved.

10.0 Adverse Events

10.1. Definitions

Adverse Events (AE) is any unfavorable and unintended sign (including an abnormal laboratory finding), symptom or disease temporally associated with the use of a medicinal (investigational) product, treatment or procedure regardless of whether it is considered related to the medical treatment or procedure *(NCI CTEP Guidelines March 28, 2011).*

Serious Adverse Event (SAE) is an AE that is fatal or life threatening, requires inpatient hospitalization or prolongation of existing hospitalization (for > 24 hours), persistent or significant incapacity or substantial disruption of the ability to conduct normal life functions, or is a congenital anomaly/ birth defect, or results in any important medical event that may not result in death, be life threatening, or require hospitalization may be considered an SAE when, based upon appropriate medical judgment, may jeopardize the subject and may require medical or surgical intervention to prevent any of the above outcomes. A "life-threatening" adverse event places the patient at immediate risk of death in the judgment of the investigator or sponsor.

10.2. Severity Rating

The investigator will evaluate the severity of each adverse event. NCI Common Terminology Criteria for Adverse Events (CTCAE v.4.03, June 2010) or study specific toxicity tables provided in the protocol define severity. If not included in CTCAE v.4.0, severity is expressed in numerical grade using the following definitions:

- Grade 1: Mild-asymptomatic or mild symptoms; clinical or diagnostic observations only; intervention not indicated.
- Grade 2: Moderate-minimal, local or noninvasive intervention indicated; limiting age appropriate instrumental ADL.
- Grade 3: Severe-severe or medically significant but not immediately life-threatening; hospitalization or prolongation of hospitalization indicated; disabling; limiting self-care ADL.
- Grade 4: Life-threatening consequences; urgent intervention indicated.
- Grade 5: Death related to AE.

10.3. Attribution/Relationship to study drug

1. Definite – clearly related
2. Probable – likely related
3. Possible – may be related
4. Unlikely – doubtfully related
5. Unrelated – clearly not related

10.4. Expectedness

An Expected Adverse Event is one where the specificity or severity is consistent with the current information available from the resources.

Copyright© 2016 Fox Chase Cancer Center® Office of Clinical Research. All rights reserved.

An Unexpected Adverse Event is one where the nature, severity, or frequency of the event is related to participation in the research is not consistent with either:

1. The known or foreseeable risk of adverse events associated with the procedures involved in the research that are described in (a) the protocol-related documents, such as the IRB-approved research protocol, any applicable investigator brochure, and the current IRB-approved informed consent document, and (b) other relevant sources of information, such as product labeling and package inserts: or

2. The expected natural progression of any underlying disease, disorder, or condition of the subject (s) experiencing the adverse event and the subjects(s) predisposing risk factor profile for the adverse event. (OHRP Guidance on reviewing unanticipated problems 2007)

10.5. Recording and Reporting Responsibilities

10.5.1. Investigative site recording responsibilities:

1. Upon identification of an AE or SAE, the site investigator will utilize the above definitions to properly classify the event. Each category listed above must be recorded for each event.

2. All AEs and SAEs will be recorded in the "AE case report forms" (CRF) and in progress reports with details about the grade and attribution of each episode, action taken with respect to the study drug, and the patient's outcome will be recorded in the CRF. The study period during which all AEs and SAEs must be reported begins at the initiation of study treatment and ends 42 days following the last administration of study treatment or study discontinuation/termination, whichever is earlier. After this period, investigators should only report SAEs that are attributed to prior study treatment. All events will be recorded on case report forms for the duration of the study until they resolve.

3. All reportable SAEs will be recorded on the FDA MedWatch form 3500a. After submitting the initial report it may be necessary to submit follow up reports should the event require further investigation.

10.5.2. Investigative site reporting responsibilities:

1. The investigator/ site is responsible to report all SAEs that occur on or after the first day of study treatment to the IST Regulatory Specialist within 24 hours of becoming aware of the event. All subsequent SAEs must be reported for up to 30 days after the last treatment.

   Each investigator is responsible to report all AEs/SAEs to their local IRB following guidelines set by that IRB. The FCCC OCR reserves the right to request an event be reported to the IRB at their discretion. Copies of events

Copyright© 2016 Fox Chase Cancer Center® Office of Clinical Research. All rights reserved.

reviewed by the IRB must be sent email to the IST Regulatory Specialist at **SAE.FCCC@fccc.edu.**

2. If the investigator or IRB feels the event warrants a revision to the informed consent that was not already initiated by the OCR, draft revisions will be made in track changes and submitted to the OCR for consideration. Any consent revisions must receive OCR approval **prior** to submission to the IRB.

3. Any investigator who is in doubt of whether a particular AE needs to be reported is directed to call the Study Monitor for confirmation with the Sponsor Investigator.

4. If the results of an investigator or OCR investigation show an adverse event not initially determined to be reportable is so reportable, the investigator will report the event following the above guidelines based on the date the determination is made.

5. Copies of all related correspondence and reporting documents must be submitted to the ISRU and will be maintained in the trial master file.

Participating sites should report events to:

Investigator-Sponsored Research Unit
Office of Clinical Research
Fox Chase Cancer Center
SAE.FCCC@fccc.edu

10.5.3. OCR Reporting Responsibilities:

1. Adverse events which meet all of the following criteria must be reported to all participating institutions for IRB submission within 2 weeks of notification of the event.

    i. Unexpected (in terms of nature, severity, or frequency) given (a) the research procedures that are described in the protocol-related documents, such as the IRB-approved research protocol and informed consent document; and (b) the characteristics of the subject population being studied;

    ii. Possibly related to participation in the research (possibly related means there is a reasonable possibility that the incident, experience, or outcome may have been caused by the procedures involved in the research); and

Copyright© 2016 Fox Chase Cancer Center® Office of Clinical Research. All rights reserved.

iii. Serious (refer to above definition) or otherwise one that suggests that the research places subjects or others at a greater risk of physical or psychological harm than was previously known or recognized.

2. If the adverse event requires modification of the study protocol and informed consent, these changes will be provided to all participating institutions in the form of an amendment from the ISRU for each site's IRB of record along with the report of the adverse event.

3. Copies of all related correspondence and reporting documents will be maintained in a centralized regulatory file for this study at OCR.

4. SAEs that are related, unexpected, fatal, or life-threatening are reportable through the Food and Drug Administration (FDA) MedWatch program by telephone or fax no later than 7 calendar days after initial receipt of the information. Further information on the timing of submissions are as directed by FDA guidelines (http://www.fda.gov/medwatch/index.html). Serious, unexpected events that suggest significant clinical risk will be submitted to within 15 calendar days after initial receipt of this information.

   Food and Drug Administration:
   Telephone 1-800-332-1088
   Fax 1-800-332-0178
   http://www.fda.gov/medwatch/report.htm

10.5.4. SAE Reporting to Horizon

The ISRU will report all SAEs to Horizon Pharma within 1 business day of the awareness date.

10.6. Pregnancy

All WOCBP should be instructed to contact the Investigator immediately if they suspect they might be pregnant (e.g., missed or late menstrual period) at any time during study participation.

In the event of a confirmed pregnancy in a patient participating in the study, the Investigator must immediately notify the Fox Chase Cancer Center Study Monitor who will notify Matthew Zibelman MD and Horizon Pharmaceuticals.

11.0 Measures of Effect

11.1. Response Evaluation Criteria in Solid Tumors (RECIST)

The Response Evaluation Criteria in Solid Tumors (RECIST 1.1) criteria will be used for objective tumor response assessment. Assessments will be performed at baseline, on C4D1 (after 3 months at the conclusion of the combination phase), and then every 3 cycles thereafter during the single agent phase. Once protocol treatment has been

Copyright© 2016 Fox Chase Cancer Center® Office of Clinical Research. All rights reserved.

completed, subjects will be assessed approximately every three months or sooner for up to one year as determined by treating clinician.

11.2. Definitions

Evaluable for adverse events: All patients will be evaluable for adverse events from the time of their first treatment with IFN-γ.

Evaluable for objective response: Only those patients who have measurable disease present at baseline, have received at least one dose of any therapy, and have had their disease re-evaluated will be considered evaluable for response. These patients will have their response classified according to the definitions stated below. (Note: Patients who exhibit objective disease progression prior to the end of cycle 1 will also be considered evaluable).

Evaluable Non-Target Disease Response: Patients who have lesions present at baseline that are evaluable, but also have lesions that do not meet the definitions of measurable disease, have received at least one cycle of therapy, and have had their disease re-evaluated, will be considered evaluable for non-target disease. The response assessment is based on the presence, absence, or unequivocal progression of the lesions.

11.3. Disease Parameters

Measurable disease: Measurable lesions are defined as those that can be accurately measured in at least one dimension (longest diameter to be recorded) as ≥20 mm by chest x-ray, as ≥10 mm with CT scan, or ≥10 mm with calipers by clinical exam. All tumor measurements must be recorded in millimeters (or decimal fractions of centimeters).

Tumor lesions that are situated in a previously irradiated area but have demonstrated clear growth since the conclusion of radiation treatment and are deemed by the treating clinician and a radiologist to represent progressive metastatic disease will be considered measurable

Malignant lymph nodes: To be considered pathologically enlarged and measurable, a lymph node must be ≥15 mm in short axis when assessed by CT scan (CT scan slice thickness recommended to be no greater than 5 mm). At baseline and in follow-up, only the short axis will be measured and followed.

Non-measurable disease: All other lesions (or sites of disease), including small lesions (longest diameter <10 mm or pathological lymph nodes with ≥ 10 to <15 mm short axis), are considered non-measurable disease. Bone lesions, leptomeningeal disease, ascites, pleural/pericardial effusions, lymphangitis cutis/pulmonitis, inflammatory breast disease, and abdominal masses (not followed by CT or MRI), are considered as non-measurable.

Copyright© 2016 Fox Chase Cancer Center® Office of Clinical Research. All rights reserved.

Cystic lesions that meet the criteria for radiographically defined simple cysts should not be considered as malignant lesions (neither measurable nor non-measurable) since they are, by definition, simple cysts.

“Cystic lesions” thought to represent cystic metastases can be considered as measurable lesions, if they meet the definition of measurability described above. However, if non-cystic lesions are present in the same patient, these are preferred for selection as target lesions.

Target lesions: All measurable lesions up to a maximum of 2 lesions per organ and 5 lesions in total, representative of all involved organs, should be identified as target lesions and recorded and measured at baseline. Target lesions should be selected on the basis of their size (lesions with the longest diameter), be representative of all involved organs, but in addition should be those that lend themselves to reproducible repeated measurements. It may be the case that, on occasion, the largest lesion does not lend itself to reproducible measurement in which circumstance the next largest lesion which can be measured reproducibly should be selected. A sum of the diameters (longest for non-nodal lesions, short axis for nodal lesions) for all target lesions will be calculated and reported as the baseline sum diameters. If lymph nodes are to be included in the sum, then only the short axis is added into the sum. The baseline sum diameters will be used as reference to further characterize any objective tumor regression in the measurable dimension of the disease.

Non-target lesions: All other lesions (or sites of disease) including any measurable lesions over and above the 5 target lesions should be identified as non-target lesions and should also be recorded at baseline. Measurements of these lesions are not required, but the presence, absence, or in rare cases unequivocal progression of each should be noted throughout follow-up.

11.4. Methods for Evaluation of Measurable Disease

All measurements should be taken and recorded in metric notation using a ruler or calipers. All baseline evaluations should be performed as closely as possible to the beginning of treatment and never more than 28 days before the beginning of the treatment.

The same method of assessment and the same technique should be used to characterize each identified and reported lesion at baseline and during follow-up. Imaging-based evaluation is preferred to evaluation by clinical examination unless the lesion(s) being followed cannot be imaged but are assessable by clinical exam.

Clinical lesions: Clinical lesions will only be considered measurable when they are superficial (e.g., skin nodules and palpable lymph nodes) and ≥10 mm diameter as assessed using calipers (e.g., skin nodules). In the case of skin lesions, documentation by color photography, including a ruler to estimate the size of the lesion, is recommended.

Copyright© 2016 Fox Chase Cancer Center® Office of Clinical Research. All rights reserved.

Chest X-Ray: Lesions on chest x-ray are acceptable as measurable lesions when they are clearly defined and surrounded by aerated lung. However, CT is preferable.

CT and MRI: This guideline has defined measurability of lesions on CT scan based on the assumption that CT slice thickness is 5 mm or less. If CT scans have slice thickness greater than 5 mm, the minimum size for a measurable lesion should be twice the slice thickness. MRI is also acceptable in certain situations (e.g. for body scans).

MRI has excellent contrast, spatial, and temporal resolution; however, there are many image acquisition variables involved in MRI, which greatly impact image quality, lesion conspicuity, and measurement. Furthermore, the availability of MRI is variable globally.

As with CT, if an MRI is performed, the technical specifications of the scanning sequences used should be optimized for the evaluation of the type and site of disease.

Furthermore, as with CT, the modality used at follow-up should be the same as was used at baseline and the lesions should be measured/assessed on the same pulse sequence. It is beyond the scope of the RECIST guidelines to prescribe specific MRI pulse sequence parameters for all scanners, body parts, and diseases. Ideally, the same type of scanner should be used and the image acquisition protocol should be followed as closely as possible to prior scans. Body scans should be performed with breath-hold scanning techniques, if possible.

FDG PET-CT: At present the low dose or attenuation correction CT portion of a combined PET-CT is not always of optimal diagnostic CT quality for use with RECIST measurements. However, if the site can document that the CT performed as part of a PET-CT is of identical diagnostic quality to a diagnostic CT (with IV and oral contrast), then the CT portion of the PET-CT can be used for RECIST measurements and can be used interchangeably with conventional CT in accurately measuring cancer lesions over time. Note, however, that the PET portion of the CT introduces additional data which may bias an investigator if it is not routinely or serially performed.

While FDG-PET response assessments need additional study, it is sometimes reasonable to incorporate the use of FDG-PET scanning to complement CT scanning in assessment of progression (particularly possible 'new' disease). New lesions on the basis of FDG-PET imaging can be identified according to the following algorithm:

Negative FDG-PET at baseline, with a positive FDG-PET at follow-up is a sign of PD based on a new lesion.

No FDG-PET at baseline and a positive FDG-PET at follow-up: If the positive FDG-PET at follow-up corresponds to a new site of disease confirmed by CT, this is PD. If the positive FDG-PET at follow-up is not confirmed as a new site of disease on CT, additional follow-up CT scans are needed to determine if there is truly progression occurring at that site (if so, the date of PD will be the date of the initial abnormal FDG-

Copyright© 2016 Fox Chase Cancer Center® Office of Clinical Research. All rights reserved.

PET scan). If the positive FDG-PET at follow-up corresponds to a pre-existing site of disease on CT that is not progressing on the basis of the anatomic images, this is not PD.

FDG-PET may be used to upgrade a response to a CR in a manner similar to a biopsy in cases where a residual radiographic abnormality is thought to represent fibrosis or scarring. The use of FDG-PET in this circumstance should be prospectively described in the protocol and supported by disease-specific medical literature for the indication. However, it must be acknowledged that both approaches may lead to false positive CR due to limitations of FDG-PET and biopsy resolution/sensitivity.

Note: A 'positive' FDG-PET scan lesion means one which is FDG avid with an uptake greater than twice that of the surrounding tissue on the attenuation corrected image.

Ultrasound: Ultrasound is not useful in assessment of lesion size and should not be used as a method of measurement. Ultrasound examinations cannot be reproduced in their entirety for independent review at a later date and, because they are operator dependent, it cannot be guaranteed that the same technique and measurements will be taken from one assessment to the next. If new lesions are identified by ultrasound in the course of the study, confirmation by CT or MRI is advised. If there is concern about radiation exposure at CT, MRI may be used instead of CT in selected instances.

Endoscopy, Laparoscopy: The utilization of these techniques for objective tumor evaluation is not advised. However, such techniques may be useful to confirm complete pathological response when biopsies are obtained or to determine relapse in trials where recurrence following complete response (CR) or surgical resection is an endpoint.

Tumor markers: Tumor markers alone cannot be used to assess response. If markers are initially above the upper normal limit, they must normalize for a patient to be considered in complete clinical response.

Cytology, Histology: These techniques can be used to differentiate between partial responses (PR) and complete responses (CR) in rare cases (e.g., residual lesions in tumor types, such as germ cell tumors, where known residual benign tumors can remain).

The cytological confirmation of the neoplastic origin of any effusion that appears or worsens during treatment when the measurable tumor has met criteria for response or stable disease is mandatory to differentiate between response or stable disease (an effusion may be a side effect of the treatment) and progressive disease.

11.5. Response Criteria

11.5.1. Evaluation of Target Lesions

Copyright© 2016 Fox Chase Cancer Center® Office of Clinical Research. All rights reserved.

Complete Response (CR): Disappearance of all target lesions. Any pathological lymph nodes (whether target or non-target) must have reduction in short axis to <10 mm.

Partial Response (PR): At least a 30% decrease in the sum of the diameters of target lesions, taking as reference the baseline sum diameters.

Progressive Disease (PD): At least a 20% increase in the sum of the diameters of target lesions, taking as reference the smallest sum on study (this includes the baseline sum if that is the smallest on study). In addition to the relative increase of 20%, the sum must also demonstrate an absolute increase of at least 5 mm. (Note: the appearance of one or more new lesions is also considered progressions).

Stable Disease (SD): Neither sufficient shrinkage to qualify for PR nor sufficient increase to qualify for PD, taking as reference the smallest sum diameters while on study

11.5.2. Evaluation of Non-Target Lesions

Complete Response (CR): Disappearance of all non-target lesions and normalization of tumor marker level. All lymph nodes must be non-pathological in size (<10 mm short axis).

If tumor markers are initially above the upper normal limit, they must normalize for a patient to be considered in complete clinical response.

Non-CR/Non-PD: Persistence of one or more non-target lesion(s) and/or maintenance of tumor marker level above the normal limits.

Progressive Disease (PD): Appearance of one or more new lesions and/or unequivocal progression of existing non-target lesions. Unequivocal progression should not normally trump target lesion status. It must be representative of overall disease status change, not a single lesion increase. Although a clear progression of “non-target” lesions only is exceptional, the opinion of the treating physician should prevail in such circumstances, and the progression status should be confirmed at a later time by the review panel (or Principal Investigator).

11.5.3. Evaluation of Best Overall Response (BOR)

The BOR is the best response recorded from the start of the treatment until disease progression/recurrence (taking as reference for progressive disease the smallest measurements recorded since the treatment started). The patient's best response assignment will depend on the achievement of both measurement and confirmation criteria.

Copyright© 2016 Fox Chase Cancer Center® Office of Clinical Research. All rights reserved.

Table 9: Determination of BOR for Patients with Measurable Disease (i.e. Target Disease)

Target Lesions	Non-Target Lesions	New Lesions	Overall Response	Best Overall Response when Confirmation is Required*
CR	CR	No	CR	≥4 wks. Confirmation**
CR	Non-CR/Non-PD	No	PR	≥4 wks. Confirmation**
CR	Not evaluated	No	PR	
PR	Non-CR/Non-PD /not evaluated	No	PR	
SD	Non-CR/Non-PD /not evaluated	No	SD	Documented at least once ≥4 wks. from baseline**
PD	Any	Yes or No	PD	no prior SD, PR or CR
Any	PD***	Yes or No	PD	
Any	Any	Yes	PD	

* See RECIST 1.1 manuscript for further details on what is evidence of a new lesion.
** Only for non-randomized trials with response as primary endpoint.
*** In exceptional circumstances, unequivocal progression in non-target lesions may be accepted as disease progression.
Note:Patients with a global deterioration of health status requiring discontinuation of treatment without objective evidence of disease progression at that time should be reported as "*symptomatic deterioration.*" Every effort should be made to document the objective progression even after discontinuation of treatment.

11.6. Duration

Duration of overall response: measured from the time measurement criteria are met for CR or PR (whichever is first recorded) until the first date that recurrent or progressive disease is objectively documented (taking as reference for progressive disease the smallest measurements recorded since the treatment started).

Duration of overall CR: measured from the time measurement criteria are first met for CR until the first date that progressive disease is objectively documented.

Duration of stable disease: measured from the start of the treatment until the criteria for progression are met, taking as reference the smallest measurements recorded since the treatment started, including the baseline measurements.

11.7. Progression-Free Survival

Defined as the duration of time from start of treatment to time of progression or death, whichever occurs first.

12.0 Statistical Considerations

12.1. Study Design/Endpoints

12.1.1. Primary Endpoint: Safety and Tolerability

Copyright© 2016 Fox Chase Cancer Center® Office of Clinical Research. All rights reserved.

Phase I patients will be treated as part of three dose levels of 6patients each in a 6 dose escalation design as described above in section 5.0. Expansion cohorts of 15 patients each as described previously will be opened at the determined RP2D.

During the dose expansion phase, early stopping rules and/or dose reductions of IFN-γ will be made as defined in section 5.0, table 5 above.

The study will be terminated after 5 expansion patients if ever 3 of them experience DLT. The chance of this occurrence is 5.8% if the true DLT rate is 20%. This chance is 31.7% if the DLT rate is 40%. Similarly the table gives the chance of termination after 8, 12, or 15 patients have been tested. The total chance of declaring the treatment too toxic, either early or after all 15 expansion patients have been tested, is 12.5% or 68.3% depending on whether the true DLT rate is 20% or 40% respectively.

Table 10 below gives the chance of termination during the expansion cohorts in terms of the rules defined in table 5, while table 11 gives the chance that the treatment will be found to be too toxic as a function of the true DLT rate.

Table 10: Chance of Study Termination of Expansion Cohorts Based on Early Termination Rules

DLTs/n	p(DLT) = 0.20	p(DLT) = 0.40
3/5	0.058	0.317
4/8	0.025	0.138
5/12	0.030	0.160
6/15	0.014	0.067
TOTAL	0.125	0.683

Table 11: Chance of Study Termination of Expansion Cohorts as Function of True DLT Rate

p(DLT) (%)	p (Declare Toxic)
10	0.014
20	0.125
30	0.384
40	0.683
50	0.889
60	0.976
70	0.998
80	0.999
90	1.000

12.1.2. Secondary Endpoint: ORR in the individual disease specific dose expansion cohorts

Copyright© 2016 Fox Chase Cancer Center® Office of Clinical Research. All rights reserved.

ORR= complete response + partial response, as determined using RECIST v1.1 criteria, and characterized using 95% confidence intervals. The ORR will be estimated as the proportion of patients meeting OR definition per total number of patients in each dose expansion cohort, plus any patients from the dose escalation portion treated at the RP2D. The ORR and its 95% confidence interval will be reported.

12.1.3. Secondary Endpoints: PFS and OS in the individual disease specific dose expansion cohorts

Kaplan-Meier (KM) curves will be used to estimate PFS, OS in the dose expansion cohorts, including any patients of the same disease type treated at the RP2D in the dose escalation portion.

12.1.4. Secondary Endpoint: OS at 1 year in the disease specific dose-expansion cohorts

OS at one year will be derived from the Kaplan-Meier curves in each disease specific expansion cohort and reported with its 95% confidence interval.

12.1.5. Analysis of Correlative Endpoints

- Expression of PD-L1 on tumor cells and immune cells in the tumor microenvironment
- Pre-treatment and on-treatment biopsy specimens will be measured using IHC and SMI methods, as described previously and in the lab manual, displayed graphically and compared using for statistically significant changes using methods such as paired t-tests
- Changes in IFN-γ related serum markers
- We will use graphical displays to evaluate changes in serum markers between pre- and post-treatment. A paired t-test may be used to determine if there is a statistically significant change
- Soluble PD-L1, and immune cell PD-1 expression

Samples from peripheral blood samples taken before, during, and after treatment will be analyzed using flow cytometry and ELISA and compared using paired t-tests

Samples obtained from tumor biopsy will be analyzed by the Nanostring nCounter® Analysis System platform for comparison to IHC and flow cytometric analyses

12.2. Sample Size/Accrual Rate

12.2.1. Planned Sample Size

<u>Dose escalation phase</u>: 18 patients, six patients at each dose level of IFN-γ, with up to three dose levels to establish the RP2D. Additional dose escalation

Copyright© 2016 Fox Chase Cancer Center® Office of Clinical Research. All rights reserved.

cohorts of 6 patients each may be added at lower doses if deemed necessary based on safety data or correlative data as outlined above.

Dose expansion phase: 15 patients in each dose expansion cohort to evaluate for biomarker and immunologic effects as well as some signal of efficacy in targeted patient populations with published sensitivity to PD-1 inhibition.

With 15 patients treated at the RP2D per cohort based on the expansions, the chance of observing at least one toxicity in each disease group when the frequency of that specific toxicity is 5%, 10% or 20% will be 54%, 79% and 96%, respectively.

Accrual Rate: 2 patients /month

Stratification Factors: none

12.3. Reporting and Exclusions

Evaluation of toxicity: All patients will be evaluable for toxicity from the time of their first treatment with IFN-γ

DLT Assessment: Evaluation will occur during the first 6 weeks of the combination phase

Evaluation for irAEs: All patients who receive nivolumab will be monitored for irAEs

Evaluation of response: All patients included in the study must be assessed for response to treatment, even if there are major protocol treatment deviations or if they are ineligible. Each patient will be assigned one of the following categories:

- Complete response
- Partial response
- Stable disease
- Progressive disease
- Early death from malignant disease
- Early death from toxicity
- Early death because of other cause
- Early death from irAE
- Unknown (not assessable, insufficient data)

Note: All of the patients who met the eligibility criteria (with the possible exception of those who received no study medication) should be included in the main analysis of the response rate. Patients in response categories 4-9 should be considered to have a treatment failure (disease progression). Thus, an incorrect treatment schedule or drug administration does not result in exclusion from the analysis of the response rate. Precise definitions for categories 4-9 will be protocol specific.

Copyright© 2016 Fox Chase Cancer Center® Office of Clinical Research. All rights reserved.

13.0 Data and Safety Monitoring Plan

13.1. Monitoring Plan

FCCC ISRU will monitor the medical and study records of each participant accrued throughout the course of the study. In addition, the ISRU will collect and report data to the Sponsor-Investigator who will review these data on a real time basis at a rate dependent on subject accrual. All serious adverse events (SAEs) will be reviewed on a real time basis first by the study site PI and subsequently by the CTO and study PI as applicable.

13.2. Data Safety Monitoring Board

Interim analysis of toxicity, outcome and ongoing scientific investigations will be performed by the Fox Chase Cancer Center Data Safety Monitoring Board (FCCC DSMB). In this capacity the FCCC DSMB will serve as an advisory committee to the Sponsor-Investigator. The FCCC DSMB will review those aspects of this trial that are outlined in the responsibilities section of the Data and Safety Monitoring Plan (DSMP). If the committee decides that changes should be made to this trial, it will make recommendations in writing to the Sponsor Investigator, the Associate Director of Clinical Research, and the Protocol Management Executive Committee, which, in turn, have the authority to approve or disapprove these recommendations. These changes will be discussed with the Sponsor-Investigator before they are implemented. These changes may include early termination of accrual. Other changes might include altering the accrual goals or changing the eligibility criteria for the trial.

14.0 Administrative

This study will be conducted in accordance will local, state and Federal regulations and according to accepted good clinical practice guidelines.

14.1. Data Reporting

The FCCC Study Monitor will request case report forms to be completed within 2 weeks of the protocol visit. Participating sites are responsible to respond to queries prior to the next scheduled monitoring visit.

The ISRU is responsible for compiling and submitting data to the Sponsor-Investigator and statistician on an ongoing basis for monitoring as described in the data safety monitoring plan and reporting to the Extramural Data and Safety Monitoring Board.

All patient information will be stored in an EDC system accessible only to the study team members for the purpose of entering, reviewing and analyzing data. Any paper records, such as case report files, produced will be stored in a secure location.

The ISRU is responsible for distributing and tracking review of all IND Action Letters, Safety Reports, study specific Serious Adverse Events

Copyright© 2016 Fox Chase Cancer Center® Office of Clinical Research. All rights reserved.

14.2. Retention of Records

Time points for the retention of records are described in detail in the contract between the grantor and the OCR and passed on to the participating site. Please refer to the study specific terms for specific time points. In all cases the Study Monitor must be notified of any plans to move records to an offsite location prior to doing so.

14.3. Study Agents

Any study agent supplied through the OCR from the manufacturer or a third party distributor may not be used for any purpose outside the scope of this protocol. The agent may not be transferred to any party not participating in the clinical trial.

14.4. Informed Consent

The IRB approved informed consent documents must be signed by the patient, or the patient's legally authorized representative, before his or her participation in the study. The case history for each patient shall document that informed consent was obtained prior to participation in the study. A copy of the informed consent documents must be provided to the patient or the patient's legally authorized representative. If applicable, they will be provided in a certified translation of the local language.

Original signed consent forms must be filed in each patient's study file or medical record with a copy in the study file.

Copyright© 2016 Fox Chase Cancer Center® Office of Clinical Research. All rights reserved.

15.0 References

References

1. Siegel, R., et al., *Cancer statistics, 2014.* CA Cancer J Clin, 2014. **64**(1): p. 9-29.
2. *SEER Stat Fact Sheets: Kidney and Renal Pelvis*. 2003-2009.
3. Gupta, K., et al., *Epidemiologic and socioeconomic burden of metastatic renal cell carcinoma (mRCC): a literature review.* Cancer treatment reviews, 2008. **34**(3): p. 193-205.
4. Fyfe, G., et al., *Results of treatment of 255 patients with metastatic renal cell carcinoma who received high-dose recombinant interleukin-2 therapy.* J Clin Oncol, 1995. **13**(3): p. 688-96.
5. Negrier, S., et al., *Recombinant human interleukin-2, recombinant human interferon alfa-2a, or both in metastatic renal-cell carcinoma. Groupe Francais d'Immunotherapie.* N Engl J Med, 1998. **338**(18): p. 1272-8.
6. McDermott, D.F., et al., *Randomized phase III trial of high-dose interleukin-2 versus subcutaneous interleukin-2 and interferon in patients with metastatic renal cell carcinoma.* J Clin Oncol, 2005. **23**(1): p. 133-41.
7. Coppin, C., et al., *Immunotherapy for advanced renal cell cancer.* Cochrane Database Syst Rev, 2005(1): p. CD001425.
8. Motzer, R.J., et al., *Pazopanib versus sunitinib in metastatic renal-cell carcinoma.* N Engl J Med, 2013. **369**(8): p. 722-31.
9. Pardoll, D.M., *The blockade of immune checkpoints in cancer immunotherapy.* Nat Rev Cancer, 2012. **12**(4): p. 252-64.
10. Dunn, G.P., et al., *Cancer immunoediting: from immunosurveillance to tumor escape.* Nat Immunol, 2002. **3**(11): p. 991-8.
11. Brahmer, J.R., et al., *Phase I study of single-agent anti–programmed death-1 (MDX-1106) in refractory solid tumors: safety, clinical activity, pharmacodynamics, and immunologic correlates.* Journal of Clinical Oncology, 2010. **28**(19): p. 3167-3175.
12. Brahmer, J.R., et al., *Safety and activity of anti-PD-L1 antibody in patients with advanced cancer.* N Engl J Med, 2012. **366**(26): p. 2455-65.
13. Hamid, O., et al., *Safety and tumor responses with lambrolizumab (anti–PD-1) in melanoma.* New England Journal of Medicine, 2013. **369**(2): p. 134-144.
14. Topalian, S.L., et al., *Safety, activity, and immune correlates of anti–PD-1 antibody in cancer.* New England Journal of Medicine, 2012. **366**(26): p. 2443-2454.
15. Weber, J.S., K.C. Kähler, and A. Hauschild, *Management of immune-related adverse events and kinetics of response with ipilimumab.* Journal of Clinical Oncology, 2012. **30**(21): p. 2691-2697.
16. Motzer, R.J., et al., *Nivolumab for Metastatic Renal Cell Carcinoma: Results of a Randomized Phase II Trial.* Journal of Clinical Oncology, 2014: p. JCO. 2014.59. 0703.
17. Howlader, N., et al. *SEER Cancer Statistics Review, 1975-2011*. 2014 [cited 2015 January 14]; Available from: http://seer.cancer.gov/csr/1975_2011/.
18. Askeland, E.J., et al., *Bladder Cancer Immunotherapy: BCG and Beyond.* Adv Urol, 2012. **2012**: p. 181987.
19. Joudi, F.N., et al., *Final results from a national multicenter phase II trial of combination bacillus Calmette-Guerin plus interferon alpha-2B for reducing recurrence of superficial bladder cancer.* Urol Oncol, 2006. **24**(4): p. 344-8.

Copyright© 2016 Fox Chase Cancer Center® Office of Clinical Research. All rights reserved.

20. Miller, C.H., S.G. Maher, and H.A. Young, *Clinical Use of Interferon-γ.* Annals of the New York Academy of Sciences, 2009. **1182**(1): p. 69-79.
21. Giannopoulos, A., et al., *The immunomodulating effect of interferon-γ intravesical instillations in preventing bladder cancer recurrence.* Clinical cancer research, 2003. **9**(15): p. 5550-5558.
22. von der Maase, H., et al., *Gemcitabine and cisplatin versus methotrexate, vinblastine, doxorubicin, and cisplatin in advanced or metastatic bladder cancer: results of a large, randomized, multinational, multicenter, phase III study.* Journal of Clinical Oncology, 2000. **18**(17): p. 3068-3077.
23. von der Maase, H., et al., *Long-term survival results of a randomized trial comparing gemcitabine plus cisplatin, with methotrexate, vinblastine, doxorubicin, plus cisplatin in patients with bladder cancer.* Journal of Clinical Oncology, 2005. **23**(21): p. 4602-4608.
24. Plimack, E., et al., *LBA23A PHASE 1B STUDY OF PEMBROLIZUMAB (PEMBRO; MK-3475) IN PATIENTS (PTS) WITH ADVANCED UROTHELIAL TRACT CANCER.* Annals of Oncology, 2014. **25**(suppl 4): p. mdu438. 24.
25. Bellmunt, J., et al., *808OINHIBITION OF PD-L1 BY MPDL3280A LEADS TO CLINICAL ACTIVITY IN PTS WITH METASTATIC UROTHELIAL BLADDER CANCER (UBC).* Annals of Oncology, 2014. **25**(suppl 4): p. iv280-iv280.
26. Powles, T., et al., *MPDL3280A (anti-PD-L1) treatment leads to clinical activity in metastatic bladder cancer.* Nature, 2014. **515**(7528): p. 558-62.
27. Farrar, M.A. and R.D. Schreiber, *The molecular cell biology of interferon-gamma and its receptor.* Annual review of immunology, 1993. **11**(1): p. 571-611.
28. Gleave, M.E., et al., *Interferon gamma-1b compared with placebo in metastatic renal-cell carcinoma.* New England Journal of Medicine, 1998. **338**(18): p. 1265-1271.
29. Kleeberg, U., et al., *Final results of the EORTC 18871/DKG 80-1 randomised phase III trial: rIFN-α2b versus rIFN-γ versus ISCADOR M® versus observation after surgery in melanoma patients with either high-risk primary (thickness> 3 mm) or regional lymph node metastasis.* European Journal of Cancer, 2004. **40**(3): p. 390-402.
30. Devane, J.G., M.L. Martin, and M.A. Matson, *A short 2 week dose titration regimen reduces the severity of flu-like symptoms with initial interferon gamma-1b treatment.* Curr Med Res Opin, 2014. **30**(6): p. 1179-87.
31. Maluish, A.E., et al., *The determination of an immunologically active dose of interferon-gamma in patients with melanoma.* J Clin Oncol, 1988. **6**(3): p. 434-45.
32. Ltd., V.T.R., *Actimmune (Interferon gamma-1b) Full Prescribing Information*. 2013 (last updated).
33. Weber, J.S., et al., *Nivolumab versus chemotherapy in patients with advanced melanoma who progressed after anti-CTLA-4 treatment (CheckMate 037): a randomised, controlled, open-label, phase 3 trial.* Lancet Oncol, 2015. **16**(4): p. 375-84.
34. Motzer, R.J., et al., *Nivolumab versus everolimus in advanced renal-cell carcinoma.* New England Journal of Medicine, 2015.
35. Squibb, B.-M., *Nivolumab Full Prescribing Information*. 2015.
36. Powles, T., et al., *MPDL3280A (anti-PD-L1) treatment leads to clinical activity in metastatic bladder cancer.* Nature, 2014. **515**(7528): p. 558-562.
37. Robert, C., et al., *Pembrolizumab versus ipilimumab in advanced melanoma.* New England Journal of Medicine, 2015.

Copyright© 2016 Fox Chase Cancer Center® Office of Clinical Research. All rights reserved.

38. Borghaei, H., et al., *Nivolumab versus Docetaxel in Advanced Nonsquamous Non-Small-Cell Lung Cancer.* N Engl J Med, 2015.
39. Brahmer, J., et al., *Nivolumab versus docetaxel in advanced squamous-cell non–small-cell lung cancer.* New England Journal of Medicine, 2015. **373**(2): p. 123-135.
40. Ferris, R.L., et al., *Nivolumab for recurrent squamous-cell carcinoma of the head and neck.* New England Journal of Medicine, 2016.
41. Ansell, S.M., et al., *PD-1 blockade with nivolumab in relapsed or refractory Hodgkin's lymphoma.* New England Journal of Medicine, 2015. **372**(4): p. 311-319.
42. Garon, E.B., et al., *Pembrolizumab for the Treatment of Non–Small-Cell Lung Cancer.* New England Journal of Medicine, 2015.
43. Seiwert, T.Y., et al. *Antitumor activity and safety of pembrolizumab in patients (pts) with advanced squamous cell carcinoma of the head and neck (SCCHN): Preliminary results from KEYNOTE-012 expansion cohort*. in *ASCO Annual Meeting Proceedings*. 2015.
44. Rosenberg, J.E., et al., *Atezolizumab in patients with locally advanced and metastatic urothelial carcinoma who have progressed following treatment with platinum-based chemotherapy: a single-arm, multicentre, phase 2 trial.* The Lancet, 2016.
45. Fehrenbacher, L., et al., *Atezolizumab versus docetaxel for patients with previously treated non-small-cell lung cancer (POPLAR): a multicentre, open-label, phase 2 randomised controlled trial.* The Lancet, 2016. **387**(10030): p. 1837-1846.
46. Rittmeyer, A., et al., *Atezolizumab versus docetaxel in patients with previously treated non-small-cell lung cancer (OAK): a phase 3, open-label, multicentre randomised controlled trial.* The Lancet, 2016.
47. Administration, U.F.a.D. *Approved Drugs- Pembrolizumab*. 2014 [cited 2014 September 4]; Available from: http://www.fda.gov/Drugs/InformationOnDrugs/ApprovedDrugs/ucm412861.htm;.
48. Administration, U.F.a.D. *Approved Drugs- Nivolumab*. 2014 [cited 2014 December 22]; Available from: http://www.fda.gov/NewsEvents/Newsroom/PressAnnouncements/ucm427716.htm.
49. Squibb, B.-M., *Study of BMS-936558 (Nivolumab) compared to docetaxel in previously treated advanced or metastatic squamous cell non-small cell lung cancer (NSCLC)(CheckMate 017).* clinicaltrials. gov/show/NCT01642004, 2013.
50. Taube, J.M., et al., *Association of PD-1, PD-1 ligands, and other features of the tumor immune microenvironment with response to anti-PD-1 therapy.* Clinical Cancer Research, 2014: p. clincanres. 3271.2013.
51. Gerlinger, M., et al., *Intratumor heterogeneity and branched evolution revealed by multiregion sequencing.* New England Journal of Medicine, 2012. **366**(10): p. 883-892.
52. Pardoll, D. and C. Drake, *Immunotherapy earns its spot in the ranks of cancer therapy.* J Exp Med, 2012. **209**(2): p. 201-9.
53. Taube, J.M., et al., *Association of PD-1, PD-1 ligands, and other features of the tumor immune microenvironment with response to anti–PD-1 therapy.* Clinical Cancer Research, 2014. **20**(19): p. 5064-5074.
54. Dong, H., et al., *Tumor-associated B7-H1 promotes T-cell apoptosis: a potential mechanism of immune evasion.* Nature medicine, 2002. **8**(8): p. 793-800.
55. Eppihimer, M.J., et al., *Expression and regulation of the PD-L1 immunoinhibitory molecule on microvascular endothelial cells.* Microcirculation, 2002. **9**(2): p. 133-145.

Copyright© 2016 Fox Chase Cancer Center® Office of Clinical Research. All rights reserved.

56. Lee, S.J., et al., *Interferon regulatory factor-1 is prerequisite to the constitutive expression and IFN-gamma-induced upregulation of B7-H1 (CD274).* FEBS Lett, 2006. **580**(3): p. 755-62.
57. Taube, J.M., et al., *Colocalization of inflammatory response with B7-h1 expression in human melanocytic lesions supports an adaptive resistance mechanism of immune escape.* Sci Transl Med, 2012. **4**(127): p. 127ra37.
58. Mimura, K., et al., *Inhibition of mitogen-activated protein kinase pathway can induce upregulation of human leukocyte antigen class I without PD-L1-upregulation in contrast to interferon-gamma treatment.* Cancer Sci, 2014. **105**(10): p. 1236-44.
59. MacFarlane, A.W., et al., *PD-1 expression on peripheral blood cells increases with stage in renal cell carcinoma patients and is rapidly reduced after surgical tumor resection.* Cancer immunology research, 2014. **2**(4): p. 320-331.
60. Piha-Paul, S.A., et al. *Pembrolizumab (MK-3475) for patients (pts) with advanced esophageal carcinoma: Preliminary results from KEYNOTE-028*. in *ASCO Annual Meeting Proceedings*. 2015.
61. Bang, Y.-J., et al. *Relationship between PD-L1 expression and clinical outcomes in patients with advanced gastric cancer treated with the anti-PD-1 monoclonal antibody pembrolizumab (MK-3475) in KEYNOTE-012*. in *ASCO Annual Meeting Proceedings*. 2015.
62. Kang, y., et al., *Nivolumab (ONO-4538/BMS-936558) as salvage treatment after second or later-line chemotherapy for advanced gastric or gastro-esophageal junction cancer (AGC): A double-blinded, randomized, phase III trial*, in *2017 Gastrointestinal Cancers Symposium*. 2017: San Francisco, CA.
63. Wolchok, J.D., et al., *Guidelines for the evaluation of immune therapy activity in solid tumors: immune-related response criteria.* Clinical Cancer Research, 2009. **15**(23): p. 7412-7420.

Copyright© 2016 Fox Chase Cancer Center® Office of Clinical Research. All rights reserved.
