# Supplementary material for: A phase 1 study of nivolumab in combination with interferon-gamma for patients with advanced solid tumors
Source: Nat Commun. 2023 Jul 27;14:4513. doi: 10.1038/s41467-023-40028-z (PMC10374608; doi:10.1038/s41467-023-40028-z)
Supplement: Supplementary file 3 — Reporting Summary [file 41467_2023_40028_MOESM3_ESM.pdf]

nature portfolio

Corresponding author(s): Matthew Zibelman

Last updated by author(s): May 5, 2023

Reporting Summary

Nature Portfolio wishes to improve the reproducibility of the work that we publish. This form provides structure for consistency and transparency in reporting. For further information on Nature Portfolio policies, see our Editorial Policies and the Editorial Policy Checklist.

Statistics

For all statistical analyses, confirm that the following items are present in the figure legend, table legend, main text, or Methods section.

n/a	Confirmed	
☐	☒	The exact sample size (*n*) for each experimental group/condition, given as a discrete number and unit of measurement
☐	☒	A statement on whether measurements were taken from distinct samples or whether the same sample was measured repeatedly
☐	☒	The statistical test(s) used AND whether they are one- or two-sided<br>*Only common tests should be described solely by name; describe more complex techniques in the Methods section.*
☐	☒	A description of all covariates tested
☐	☒	A description of any assumptions or corrections, such as tests of normality and adjustment for multiple comparisons
☐	☒	A full description of the statistical parameters including central tendency (e.g. means) or other basic estimates (e.g. regression coefficient) AND variation (e.g. standard deviation) or associated estimates of uncertainty (e.g. confidence intervals)
☐	☒	For null hypothesis testing, the test statistic (e.g. *F*, *t*, *r*) with confidence intervals, effect sizes, degrees of freedom and *P* value noted<br>*Give P values as exact values whenever suitable.*
☒	☐	For Bayesian analysis, information on the choice of priors and Markov chain Monte Carlo settings
☒	☐	For hierarchical and complex designs, identification of the appropriate level for tests and full reporting of outcomes
☐	☒	Estimates of effect sizes (e.g. Cohen's *d*, Pearson's *r*), indicating how they were calculated

Our web collection on statistics for biologists contains articles on many of the points above.

Software and code

Policy information about availability of computer code

Data collection	Patients were accrued as part of a clinical trial. Clinical data collected by study team. No software was used for data collection
Data analysis	IHC expression analysis: Ventana Benchmark Ultra: Ventana Medical Systems, Tucson, Arizona<br><br>Flow cytometry data was acquired on a BD Aria II sorter and processed using FlowJo software (BD; version 10.7).<br><br>Cytokines in plasma were analyzed by the FCCC High Throughput Screening Facility utilizing the Human Chemokine Panel 40-plex (Bio-Plex Pro, Bio-Rad cat # 171AK99MR2) following manufacturer's protocols and plates were read on a Bio-Plex 100/200 (Bio-Rad Laboratories, Hercules CA). The instrument was driven with Bio-Plex Manager (version 6.1.0727) software and data was analyzed and exported utilizing Bio-Plex Data Pro software (version 1.2.03). Computations were made using the R language.<br><br>Correlative statistical calculations were done with Matlab R2016b Statistics and Machine Learning Toolbox.

For manuscripts utilizing custom algorithms or software that are central to the research but not yet described in published literature, software must be made available to editors and reviewers. We strongly encourage code deposition in a community repository (e.g. GitHub). See the Nature Portfolio guidelines for submitting code & software for further information.

Data

Policy information about availability of data

All manuscripts must include a data availability statement. This statement should provide the following information, where applicable:

- Accession codes, unique identifiers, or web links for publicly available datasets
- A description of any restrictions on data availability
- For clinical datasets or third party data, please ensure that the statement adheres to our policy

The datasets generated during and/or analysed during the current study are included in the published article and supplementary materials in most cases, and where they are not would be available from the corresponding author on reasonable request.

Human research participants

Policy information about studies involving human research participants and Sex and Gender in Research.

Reporting on sex and gender	This small phase I study in human cancer patients included patients regardless of sex or gender. Given the small size of the study, there would not be enough power to perform any analysis or come to any conclusions based on either sex or gender. The breakdown of patients by biological sex are reported for demographic information
Population characteristics	This information is available in the demographics section in the study manuscript. Eligible patients were >18 years of age of any gender, ECOG performance status 0-1 and had a refractory cancer with evidence of immunotherapy sensitivity at the time of the study.
Recruitment	Patients were recruited by treating clinicians from a single cancer center. Potentially eligible patients were screened upon patients signing informed consent and if eligibility confirmed allowed to proceed to study protocol. There were no pre-screening procedures or programs used and investigators who felt a patient was appropriate for study would review the eligibility criteria and consent them to participate along with the research team.
Ethics oversight	The study was approved and monitored by the Fox Chase Cancer Center Institutional Review Board

Note that full information on the approval of the study protocol must also be provided in the manuscript.

Field-specific reporting

Please select the one below that is the best fit for your research. If you are not sure, read the appropriate sections before making your selection.

☒ Life sciences ☐ Behavioural & social sciences ☐ Ecological, evolutionary & environmental sciences

For a reference copy of the document with all sections, see nature.com/documents/nr-reporting-summary-flat.pdf

Life sciences study design

All studies must disclose on these points even when the disclosure is negative.

Sample size	This was a phase 1 study using a modified 3+3 design wherein each dose escalation cohort accrued up to 6 patients. DLTs were evaluated per the protocol and decisions on dose escalation and dose de-escalation followed protocol criteria. Additionally, cohort status was reviewed after each patient in a cohort completed the DLT phase by the Trial Steering Committee to assess totality of safety to make decisions on proceeding to the next cohort. The original cohort plan was for 3 cohorts of 6 pts (18 total pts) if RP2D determined, however the Steering Committee decided to open an additional dose de-escalation cohort of 6 pts given efficacy and correlative data suggesting more benefit at lower dosing.
Data exclusions	All patients who received any doses of study medication were included for safety analysis. Patients who were not able to make their first on study imaging exam due to toxicity or withdraw of consent were not assessed for efficacy. If patients came off for clinical progression due to their cancer, they could be followed for overall survival but not progression free survival.
Replication	This is a phase 1 study in human oncology patients. Patients were treated with various dose levels of interferon-gamma based on assigned cohort as described. Further study at the recommended phase 2 dose determined could be done to attempt to replicate results in the future.
Randomization	Patients were allocated based on the protocol specified design where cohorts of various doses of interferon-gamma were populated until DLTs hit or 6 pts met. See protocol for details. There was no randomization
Blinding	This was a phase I study and no blinding was incorporated so investigators could assess toxicity of the study combination.

Reporting for specific materials, systems and methods

We require information from authors about some types of materials, experimental systems and methods used in many studies. Here, indicate whether each material, system or method listed is relevant to your study. If you are not sure if a list item applies to your research, read the appropriate section before selecting a response.

Materials & experimental systems

n/a	Involved in the study
☐	☒ Antibodies
☒	☐ Eukaryotic cell lines
☒	☐ Palaeontology and archaeology
☒	☐ Animals and other organisms
☐	☒ Clinical data
☒	☐ Dual use research of concern

Methods

n/a	Involved in the study
☒	☐ ChIP-seq
☐	☒ Flow cytometry
☒	☐ MRI-based neuroimaging

Antibodies

Antibodies used	Peripheral blood was separated into plasma and peripheral blood mononuclear cells (PBMCs). PBMCs were analyzed the same day by the FCCC Immune Monitoring Facility using 12-color multi-parametric flow cytometry employing the antibody staining panel to quantify biomarkers on T, NK, and myeloid cells utilizing leukocyte sub-gating strategies. Fluorophore-conjugated antibodies were added to 1 million PBMCs in 100 µL staining buffer (Hanks's Balanced Salt Solution + 1% heat inactivated fetal bovine serum and 0.09% sodium azide), incubated on ice x 20 minutes, and washed twice with staining buffer before analysis. Surface PD-1 was measured by primary staining of all those samples with unlabeled nivolumab (1 µg in 100 µl wash buffer on ice x 20 minutes), followed by two washes, secondary staining with fluorophore-conjugated anti-human IgG4 (10 µL in 100 µL staining buffer on ice x 20 minutes), and two washes on ice.
Validation	Extensive validation confirmation is provided as part of the supplementary files and is to extensive to list here.

Clinical data

Policy information about clinical studies

All manuscripts should comply with the ICMJEguidelines for publication of clinical research and a completedCONSORT checklist must be included with all submissions.

Clinical trial registration	NCT02614456
Study protocol	Full protocol included in the submission
Data collection	The trial was performed soley at Fox Chase Cancer Center in Philadelphia, PA. Recruitment was started December 2015 and completed 2/2018 when the final patient signed consent. Data collection continued until the final patient went off study 12/2018
Outcomes	The primary endpoint was safety and was defined in the protocol. There were strict DLT criteria at each dose level and if DLT rules were triggered that cohort was held and proceeded to next cohort as per protocol. Efficacy as defined in cohort included progression free survival as assessed by on study imaging, as well as overall survival from start of treatment to death.

Flow Cytometry

Plots

Confirm that:

- ☒ The axis labels state the marker and fluorochrome used (e.g. CD4-FITC).
- ☒ The axis scales are clearly visible. Include numbers along axes only for bottom left plot of group (a 'group' is an analysis of identical markers).
- ☒ All plots are contour plots with outliers or pseudocolor plots.
- ☒ A numerical value for number of cells or percentage (with statistics) is provided.

Methodology

Sample preparation	Peripheral blood samples will be collected in heparinzed tubes 1) prior to treatment, 2) after 2 weeks of IFN- therapy, and 3) after 6 weeks of pembrolizumab therapy. Samples will undergo processing within 6 hours of collection, thereby assuring integrity of biomarkers on freshly obtained leukocytes. Fresh blood samples provide overall superior results for staining of certain markers (e.g. CD62L, CD16, PD-1, and others). Whole blood will undergo separation of plasma from peripheral blood mononuclear cells (PMBC), Ficoll centrifugation, and multiparameter flow cytometry analysis of PBMC for biomarkers on monocytes, DC, NK cells, and T cells. DNA will be prepared for potential followup by genotyping and extra PBMC and plasma will undergo cryopreservation via standard operating procedures for potential future analyses.
Instrument	BD FACS Aria II flow cytometer
Software	FlowJo software

Cell population abundance	All specimens were extracted from patients on trial and processed for suitability with rigorous monitoring to maintain staining integrity. Immune cells were readily abundant in all patients for analysis.
Gating strategy	Samples will be analyzed with the following antibody staining panel (1 million PBMC stained per tube; activation markers marked in bold): 1) subsets of T cells (effector/memory, Treg) and NK cells (CD56dim/bright) by staining with anti-CD45, CD3, CD4, CD8, CD45RA, CD62L, CD25, CD127, CD56, CD69, PD-1; 2) DC and monocytes by staining with anti-lineage (CD3/CD19/CD56), CD45, HLA-DR, CD69, TREM-1, CD14, CD16, CD11c, CD123, CD86, PD-L1, PD-L2, and 3) cytolytic granules and proliferation by fix/permeabilization staining with CD45, CD3, CD4, CD8, CD56, STAT1, pSTAT1, perforin, granzyme B, and Ki67. Propidium iodide (PI) will be added to non-permeabilized samples to gate viable cells and isotype control Abs used throughout

☒ Tick this box to confirm that a figure exemplifying the gating strategy is provided in the Supplementary Information.
